# Uncovering the Cultivable Microbial Diversity of Costa Rican Beetles and Its Ability to Break Down Plant Cell Wall Components

**DOI:** 10.1371/journal.pone.0113303

**Published:** 2014-11-20

**Authors:** Gabriel Vargas-Asensio, Adrian Pinto-Tomas, Beatriz Rivera, Myriam Hernandez, Carlos Hernandez, Silvia Soto-Montero, Catalina Murillo, David H. Sherman, Giselle Tamayo-Castillo

**Affiliations:** 1 Instituto Nacional de Biodiversidad (INBio), Santo Domingo de Heredia, Costa Rica; 2 Departamento de Bioquímica, Universidad de Costa Rica, San Pedro de Montes de Oca, Costa Rica; 3 Centro de Investigación en Estructuras Microscópicas (CIEMIC), Universidad de Costa Rica, San Pedro de Montes de Oca, Costa Rica; 4 Life Sciences Institute, University of Michigan, Ann Arbor, Michigan, United States of America; 5 Escuela de Química & Centro de Investigación en Productos Naturales (CIPRONA), Universidad de Costa Rica, San Pedro de Montes de Oca, Costa Rica; Universidade Federal do Rio de Janeiro, Brazil

## Abstract

Coleopterans are the most diverse insect order described to date. These organisms have acquired an array of survival mechanisms through their evolution, including highly efficient digestive systems. Therefore, the coleopteran intestinal microbiota constitutes an important source of novel plant cell wall-degrading enzymes with potential biotechnological applications. We isolated and described the cultivable fungi, actinomycetes and aerobic eubacteria associated with the gut of larvae and adults from six different beetle families colonizing decomposing logs in protected Costa Rican ecosystems. We obtained 611 isolates and performed phylogenetic analyses using the ITS region (fungi) and 16S rDNA (bacteria). The majority of fungal isolates belonged to the order Hypocreales (26% of 169 total), while the majority of actinomycetes belonged to the genus *Streptomyces* (86% of 241 total). Finally, we isolated 201 bacteria spanning 19 different families belonging into four phyla: Firmicutes, α, β and γ-proteobacteria. Subsequently, we focused on microbes isolated from Passalid beetles to test their ability to degrade plant cell wall polymers. Highest scores in these assays were achieved by a fungal isolate (*Anthostomella* sp.), two *Streptomyces* and one *Bacillus* bacterial isolates. Our study demonstrates that Costa Rican beetles harbor several types of cultivable microbes, some of which may be involved in symbiotic relationships that enable the insect to digest complex polymers such as lignocellulose.

## Introduction

Insects are one of the most diverse groups of organisms in nature, with the ability to feed on almost any terrestrial food source. Among insects, Coleopterans, commonly called beetles, are the most diverse order. Of the approximate 300,000 reported beetle species, about 10% can be found in Costa Rica, where they inhabit an array of ecosystems with various conditions of temperature and precipitation across the whole country [1]. This versatility is partly due to their behavior, wide diversity of habitats, their capacity to generate massive numbers of offspring, and their ability to establish a variety of symbiotic relationships with microorganisms colonizing their intestines [2], [3], [4].

The insect gut represents a confined environment comprising a diverse and complex microbiota including resident and transient bacteria, actinomycetes, archaea and fungi, and it has a microbial density estimated between 10^9^–10^11^ cells/ml [5]. The gut microbiota plays an essential role in insect growth, development, nutrition, and key processes such as nitrogen fixation, pheromone production, pathogenesis and adaptability to the environment [2], [6]. This microbiota has acquired different abilities in order to establish symbiotic relationships with its host, including genomic plasticity and producing a vast diversity of enzymes [7], [8], [9].

Beetles acquire their microbiota mainly from the materials on which they feed; many of these microorganisms could have adapted to the gut environment and over time, developed mutually beneficial relationships with their host [10], [11]. This beneficial microbiota can be vertically transmitted from parents to offspring, a phenomenon that has been documented in insects such as aphids and whiteflies, but not fully characterized in beetles [12], [13], [14]. Vertical transmission of beneficial microbes may be particularly important to the Passalidae family, given that these beetles exhibit a subsocial behavior in which adults provide care for larvae sharing the same decomposing log, and even two or more generations can overlap in the same niche [11], [15].

From a biotechnology perspective, studying the cultivable microbiota colonizing beetle guts in tropical ecosystems constitutes an important, yet unexplored tool for the discovery of novel enzymes for processes including industrial production of enzymes through fermentations, lignocellulose hydrolysis, acetogenesis or methane and nitrogen fixation, all of them readily scaled in bioreactors [2]. For example, previous research has shown that the cellulolytic activity in wood-feeding beetles, especially in larval instars, is performed by microorganisms, generating several fermentation products that act as readily available energy sources, allowing the immature instars to quickly gain biomass [3], [10], [16], [17].

In order to identify microorganisms adaptable to conditions employed in biotechnological applications we explored the cultivable microbiota of beetles from Costa Rican protected ecosystems. After a survey of gut microbial diversity from several beetle genera that thrive on decomposing wood as food source, we focused on microbial isolates from Passalid beetles for in depth analyses of their plant cell wall degrading capabilities. We chose these beetles as a model because their sub-social behavior suggests that beneficial microbial transmission between adults and larvae may play an important role in their ability to degrade the recalcitrant polymers that constitute their main food source. This is supported by decades old research showing that Passalid larvae cannot survive without their parents [18], perhaps because they need to vertically acquire key microbial symbionts during development, as shown for termites [19]. Further, their behavior allows sampling both adults and larvae on the same log, thus enabling comparisons of their gut microbial community. We found that Passalid beetles gut microbiota includes a variety of cultivable microbes able to break down different plant cell wall components. Further research describing the enzymatic capabilities of these microorganisms will help understand their role in their host ecophysiology as well as their value in biotechnological and bioenergy applications.

## Materials and Methods

### Isolation of microorganisms

We collected insect specimens during five field trips to the following National Parks in Costa Rica: Carara, Puntarenas Province (9° 22′ 32″ N, 84° 9′ 9″ W); Caño Island Biological Reserve, 20 km from the Costa Rican Pacific coast, Puntarenas Province (8° 42′ 0″ N, 83° 52′ 0″ W); Arenal Volcano in Guanacaste province (10° 27′ 48″ N, 84° 42′ 12″ W) and two in Braulio Carrillo, Quebrada González sector, Heredia Province (10° 9′ 36″ N, 83° 58′ 28″ W). Additionally, we collected samples at the private reserve of the Costa Rican National Biodiversity Institute (INBio) in Limon province (10° 11′ 28 N, 83° 51′ 28″ W). Sampling was performed under the collection permits of the Technical Office of CONAGEBIO (Ministry of Environment and Telecommunications), Costa Rica. We sampled the interior of decomposing logs and collected larvae and adults from six different beetle families: Passalidae, Staphylinidae, Scarabaeidae, Cerambycidae, Tenebrionidae and Elateridae. All insects were transported to the laboratory at room temperature inside vented polyethylene containers and surrounded by the woody material in which they were collected (hereafter referred as gallery material). Beetle taxonomic identification was carried out by experts at INBio.

Beetles were dissected in sterile conditions, using scissors and forceps to extract the digestive tract. Gut tissues were placed in 1.5 ml tubes containing phosphate buffered saline solution, mixed in a vortex for 10 seconds and sonicated for 30 seconds. Then the homogenized gut tissues were diluted 10,000 fold with PBS solution and plated to Potato Dextrose Agar (PDA) [20] for the isolation of fungi, ISP2 Agar [21] and Chitin Agar [22] for actinomycetes; and Luria Bertani (LB) Agar for other aerobic eubacteria [20]. All plates were incubated at 28°C for up to three weeks. Isolated single colonies were transferred to fresh plates until pure cultures were obtained, which were then stored at −70°C in 20% glycerol.

### Genomic DNA isolation and Polymerase Chain Reaction (PCR)

Genomic DNA was obtained from pure microbial cultures. DNA isolations from fungi were performed by employing Nucleospin Plant kit II (plant and fungal DNA), placing approximately 200 mg of macerated mycelium in 1.5 ml microtubes containing 400 µl of lysis buffer PL1. The tissue was disrupted mechanically in a vortex after adding 30 µl of proteinase K. Tubes were incubated at 60°C for one hour and DNA was extracted according to manufacturer's specifications. Actinomycetes were treated similarly except that DNA isolation was performed by employing 50 mg of each strain and the Qiagen DNeasy Blood & Tissue Kit, according to manufacturer's instructions. Isolation of genomic DNA from eubacteria was performed using the same approach described for actinomycetes but only for strains whose 16S rRNA gene could not be amplified by direct colony PCR.

PCR was performed to amplify the Internal Transcribed Spacer (ITS) region of the 18S rRNA gene from isolated fungal strains using genomic DNA as template. We used ITS-1 as forward primer and ITS-4 as reverse primer [23]. Amplification was performed under the following conditions: initial denaturation at 94°C for 5 min; 35 cycles of 94°C for 1 min, 52°C for 1 min, 72°C for 2 min; and a final elongation step at 72°C for 10 min. The 16S rDNA genes of bacteria and actinomycetes were amplified using universal primers 27F and 1492R [24]. Amplification was performed under the following conditions: initial denaturation at 94°C for 5 min; 35 cycles of 94°C for 45 sec, 52°C for 1 min, 72°C for 90 sec; and a final elongation step at 72°C for 10 min. PCR products were purified using the following commercial kits: Qiagen QIAquick PCR Purification and Sigma-Aldrich Gen Elute PCR Clean-up kit (both according to manufacturer's protocol). Clean PCR products were sent to the Dana-Farber/Harvard Cancer Center DNA Resource Core at Harvard University (Boston MA) for sequencing. Sequences were deposited at GenBank under accession numbers: KM242228 - KM242584.

### Phylogenetic analyses

All sequences were analyzed and manually edited using SeqMan Pro-DNA STAR (http://www.dnastar.com/t-products-seqman-ngen.aspx) and MEGA 5 [25]. The identification of microorganisms was performed by comparing the sequences with those available in the GenBank database using BLAST [26]. Once edited, the sequences were aligned using GUIDANCE [27]. Identical sequences were removed from the subsequent analysis (the number of sequences removed is indicated between brackets next to the consensus sequence on the phylogenetic trees).

Phylogenetic analyses were performed using the Bayesian method with MrBayes 3.2 software [28], [29] and determining the best nucleotide substitution model using jModeltest [30], [31]. Bayesian analyses were performed for two million generations with a selection step every 1000 generations, employing four independent chains, one cold and three hot (Temperature  = 0.1) to calculate posterior probabilities for each branch (pre-set fixed option was used for prior probabilities). The consensus trees were calculated by removing the first 20,000 trees generated for higher fidelity.

### Enzyme activity test

A total of 282 isolates from Passalid beetles, including 89 fungi, 75 bacteria and 127 actinomycetes, were grown in different culture media to evaluate their enzymatic activity.

#### Cellulose hydrolysis

Carboxymethyl Cellulose (CMC) agar [32] was employed to detect cellulose hydrolysis. After microbial growth was observed, CMC plates were stained with 0.5% Congo-Red for 40 minutes and rinsed with 1 M NaCl to fix coloration. Since Congo-Red binds to cellulose, decolorized wells indicate microbial cellulose degradation [33].

#### Ligninolytic activity

MEA-Remazol Brilliant Blue R (MEA-RBBR) [32] was employed to evaluate ligninolytic activity. RBBR degradation in discolored MEA-RBBR plates was considered representative of microbial ligninolytic activity [32].

#### Lignin oxidation

A standard medium for microbial culturing was employed in these assays including, PDA for fungi (Difco, BD, Ref213400), LBA for bacteria (Invitrogen, Lennox L agar, cat227700-041) and ISP2 for actinomycetes (10 g/l Malt extract Bacto, BD, Ref218630; 4 g/l Yeast extract Bacto, BD, Ref212750; 4 g/l Dextrose J.T. Baker, 1916-01; 18 g/l Agar Bacto, BD, Ref214030; pH = 7). Incubations were performed at 25–30°C until growth was evident (two weeks maximum in case of slow growing actinomycetes). To determine lignin oxidation activity, a colloidal preparation of 1 mM 2,2′-azino-bis 3-ethylbenzothiazoline-6-sulphonic acid (ABTS, Sigma, A-3219) was poured over the standard media plates and incubated at 25°C for 2 days. Green coloration of the substrate demonstrated microbial laccase activity [34].

Results for the three assays were scored from “0” to “2”, with “0” meaning no activity, “1” meaning low activity (partial media discoloration or low coloration of ABTS) and “2” representing high activity (complete media discoloration or strong coloration of ABTS). Isolates with positive scores were selected for further semi-qualitative evaluation of their enzymatic activities related to plant cell wall degradation, including glucanase, xylanase and cellobiase activity.

#### Glucanase, xylanase and cellobiase assays

Selected isolates were inoculated again on the previously mentioned standard media and grown under the described conditions until healthy colonies were visible. Substrate solutions of Glucanase (4-Nitrophenyl Beta-D-glucopyranoside, Sigma, N7006), Xylanase (4-Nitrophenyl Beta-D-xylopyranoside, Sigma, N2132) and Cellobiase (4-Nitrophenyl Beta-D-cellobioside, Sigma, N5759) 0,6% w/v dissolved in 50 mM NaOAc; pH 5 were sprayed on each colony until they were completely covered. The plates were incubated at room temperature for 8 hours and monitored every 15 minutes. Substrate hydrolysis and the consequent liberation of 4-nitrophenyl phosphate (4NP) was shown by yellow coloration; therefore, isolates were scored qualitatively as “0” if no coloration occurred and “3”, “2” or “1” if substrate coloration took place in 1, 2 or more hours, respectively.

These results were incorporated into a matrix to build a heat map (tool available at: http://www.chibi.ubc.ca/matrix2png/bin/matrix2png.cgi http://www.chibi.ubc.ca/matrix2png/bin/matrix2png.cgi), which was then used to generate a hydrolytic profile of the isolates. Quantitative determination of enzyme activities was not performed.

### Statistical analysis

All statistical analyses were performed using the SPSS 15.0.1 statistical package (Released 2006, SPSS for Windows, version 15.0.1. Chicago, SPSS Inc.). Comparisons between groups were performed employing the MANOVA method with a significance criteria of <0.05.

## Results

### Isolation of Microorganisms

Fungi and bacteria were isolated from guts of 52 adults and 71 larvae spanning six families of Costa Rican beetles, including Passalidae (93 individuals), Scarabaeidae (11 individuals), Cerambycidae (8 individuals), Elateridae (5 individuals), Tenebrionidae (4 individuals) and Staphylinidae (2 individuals). Table 1 shows the distribution of isolates obtained by beetle family; as expected, 75% of the total isolates were isolated from Passalidae, which included most processed individuals, followed by Scarabaeidae with 18%. However, when considering the number of microorganisms isolated per specimen processed, we did not find significant differences (p<0.05) in the number of isolates obtained from the six families (Figure S1). In terms of the stage of beetle development, we did not find significant differences (p<0.05) between the number of isolates obtained from adults and larvae. In addition, although there were no significant differences in the number of bacterial isolates (both actinomycetes and other eubacteria) obtained from each protected area where samples were collected (p<0.05), we did obtain significantly less fungal isolates from beetles collected in Carara National Park (p<0.05). During the execution of this study we isolated 201 eubacteria, 169 fungi and 241 actinomycetes for a total of 611 microorganisms. The higher number of actinomycetes (41% of all isolates) is explained by the deliberate effort to select and isolate these microbes, motivated by their well known ability to produce a great variety of enzymes and secondary metabolites.

**Table 1:** Distribution of microbial isolates according to Coleopteran families sampled.

	Bacteria	Actinomycetes	Fungi	Total	Number of specimens
Passalidae	132	184	129	445	93
Scarabaeidae	44	36	24	104	11
Elateridae	5	7	3	15	5
Cerambicidae	3	5	1	9	8
Staphylinidae	3	9	5	17	2
Tenebrionidae	14	0	7	21	4
Total	201	241	169	611	123

### Molecular identification and phylogenetics

**Figure 1:**
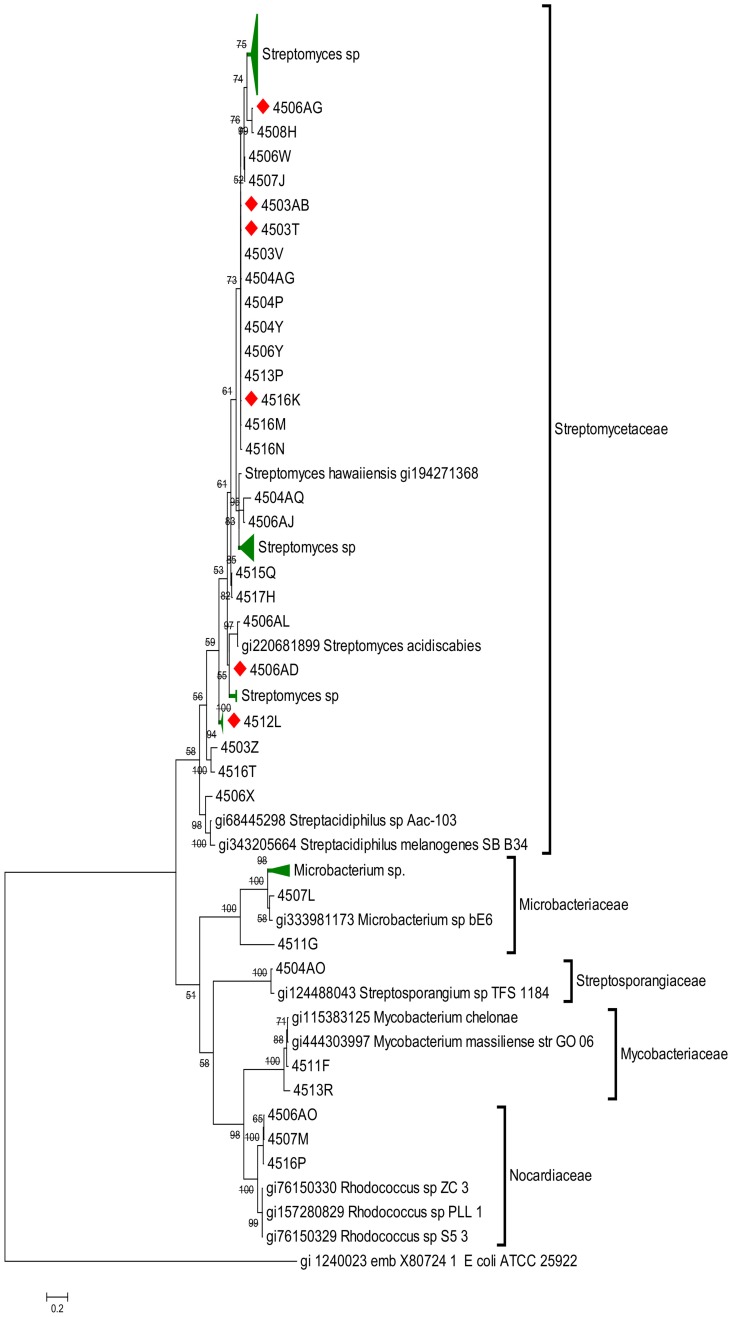
Bayesian phylogenetic tree of the rDNA 16S gene sequences of actinomycetes isolated from the gut of Costa Rican beetles. Numbers above branches represent their Bayesian-calculated posterior probabilities (two million generations, chain temperature  = 0.2, standard desviation <0.02). Compress branches are colored in green. Red diamonds indicates positive cellulolytic activity.

**Figure 2:**
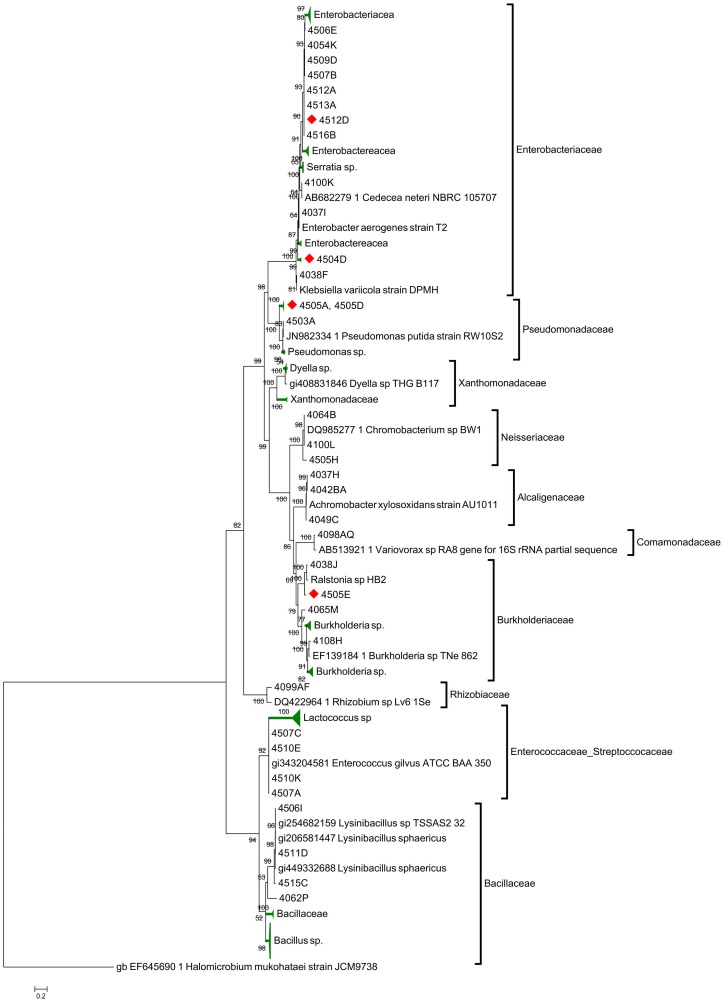
Bayesian phylogenetic tree of the rDNA 16S gene sequences of eubacteria isolated from the gut of Costa Rican beetles. Numbers above branches represent their Bayesian-calculated posterior probabilities (two million generations, chain temperature  = 0.2, standard deviation <0.02). Compress branches are colored in green. Red diamonds indicates positive cellulolytic activity.

Sequencing the 16S rDNA gene in the case of bacteria and the 18S rDNA ITS region of fungi allowed us to identify at the genus level most of the isolates obtained in this study (at least 97% of sequence homology according to BLAST identification results). The most commonly identified genus among the actinomycetes was *Streptomyces* with 86% of the isolates. Other detected genera were *Microbacterium* (3%), *Mycobacterium* (3%), *Nocardia* (2%) and *Micrococcus* (2%). Phylogenetic analyses of isolated actinomycetes showed two major clades, one for Streptomycetaeae and the other for Nocardiaceae (Figure 1). Although we found little genetic diversity, this group showed a great diversity of colony morphotypes. Bacterial isolates other than actinomycetes belonged to 13 different families and spanned four major phylogenetic classes including α, β and γ-proteobacteria and Firmicutes (Table S1). Phylogenetic analysis (Figure 2) revealed a large clade that groups all Firmicutes families, mainly Bacillaceae, Enterococcaceae and Streptococcaceae, including genera as *Bacillus*, *Paenibacillus*, *Enterococcus*, *Lactococcus* and *Lysinibacillus*. The most commonly identified genera were *Bacillus* (27%), followed by *Enterococcus* (11%). Most Gram negative isolates clade in two other major groups including, β-proteobacteria and γ-proteobacteria, with only one *Rhizobium* isolate belonging to the α-proteobacteria. The most commonly found β-proteobacteria family was Burkholderiaceae (13% of the isolates), within this group the most commonly isolated genera were *Burkholderia* (14%). Finally, among γ-proteobacteria, the Enterobacteriaceae family is the most represented with 25% of all isolates, followed by Pseudomonadaceae with 6.2%. Bacteria in the genera *Citrobacter*, *Klebsiella* and *Pseudomonas* were the most frequently isolated (Table S1).

**Figure 3:**
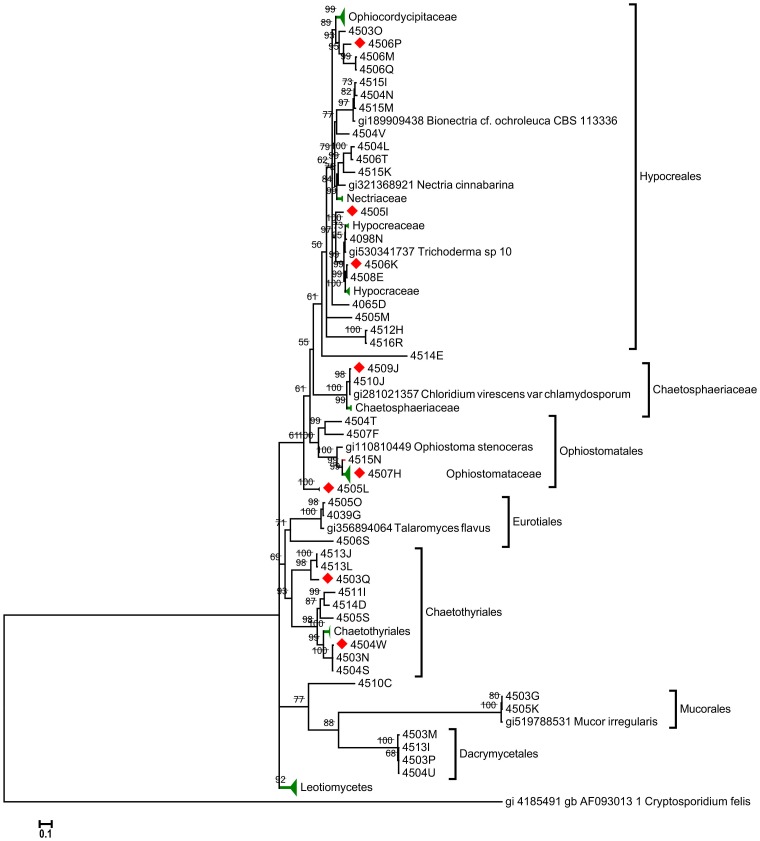
Bayesian phylogenetic tree of the ITS region sequences of fungi isolated from the gut of Costa Rican beetles. Numbers above branches represent their Bayesian-calculated posterior probabilities (two million generations, chain temperature  = 0.2, standard deviation <0.02). Numbers between brackets represent the number of isolates that shared the same sequence. Compress branches are colored in green. Red diamonds indicates positive cellulolytic activity.

The fungal isolates represented the three fungal divisions Ascomycota, Basidiomycota and Zygomycota, and showed a high diversity spanning 23 families (two of which belong to the Basidiomycota and one to Zygomycota, see Table S1). Seventy-six% of the fungal isolates were classified as ascomycetes and 18% could not be classified using the ITS region as a molecular marker. The latter are distributed in several phylogenetic groups (see Figure 3). Among the ascomycetes, the most commonly recovered group was the Hypocreales with 26% of all isolates, including the most represented genera *Trichoderma* (9% of all isolates) and *Nectria* (7%). These isolates make up the largest cluster on the fungal phylogenetic tree (Figure 3). These fungi are known for being saprophytes that are adapted to thrive in diverse environments and therefore produce a wide array of enzymes. Other Hypocreales found were *Metarhizium* and *Cordyceps*, two genera of entomopathogenic fungi in the Clavicipitaceae family (Figure 3). We also isolated fungi belonging to Ophiostomatales: the Ophiostomataceae family (10%) makes up the second largest cluster in the phylogenetic tree and were grouped next to Hypocreales. Another group of Ascomycetes detected were the Chaetosphaeriales, with 8% of all isolates belonging to this group. The Basidiomycota isolates found (2.5%) belong to the family Dacrymycetaceae. The Zigomycota isolates (2.5%), belonged to the family Mucoraceae, including the genera *Mucor* and *Rhizomucor*, which could not be separated from the Ascomycetes using the ITS as molecular marker. Finally, a significant number of fungal isolates were not identified using the ITS marker. These isolates were distributed in several phylogenetic groups (Figure 3). To identify them or to confirm that they are new species, sequencing several rRNA genes as well as select primary metabolism genes may be necessary.

### Enzymatic activity

**Figure 4:**
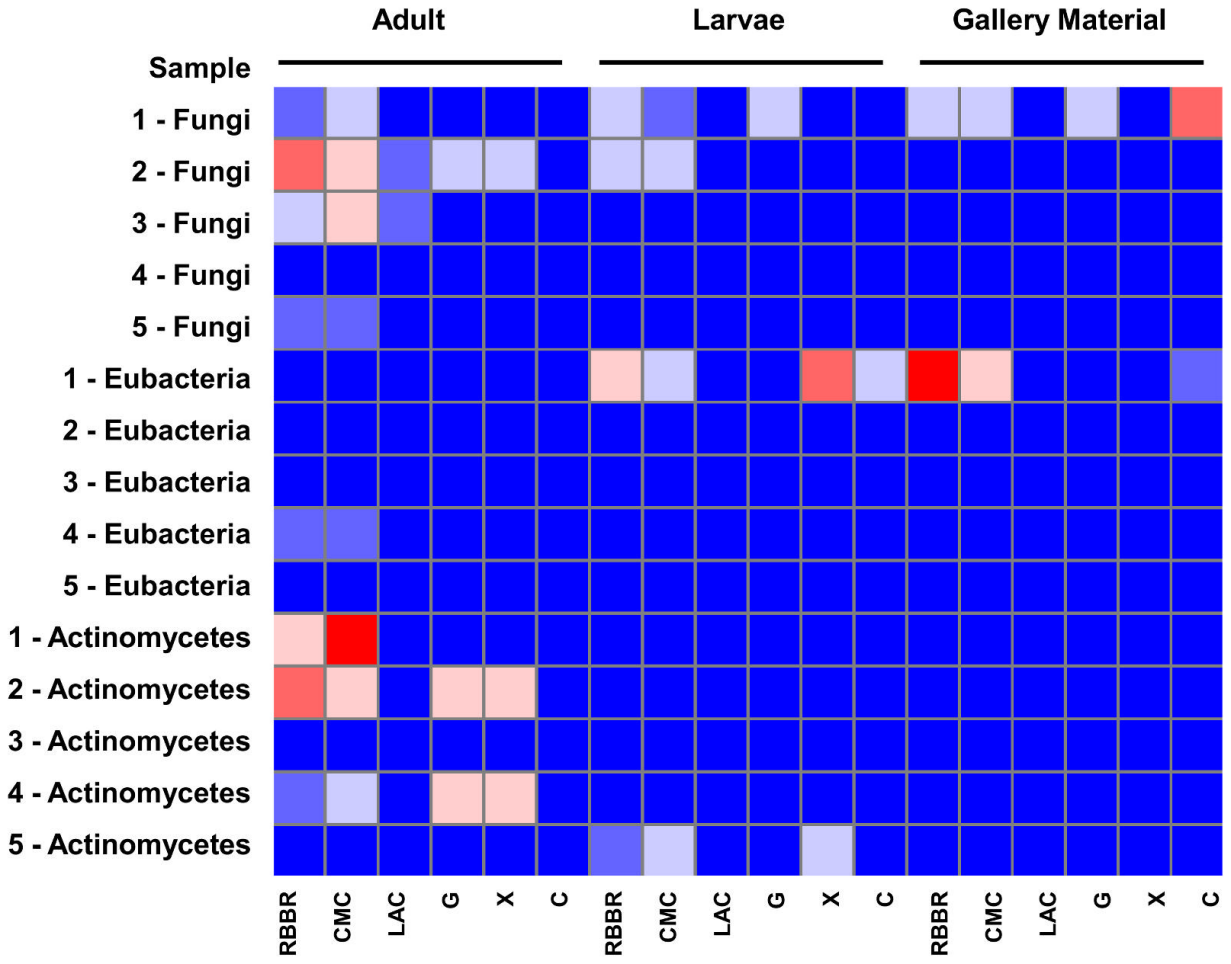
Pattern of enzymatic degradation of plant cell wall components for the fungal and bacterial isolates obtained from Passalid beetles. Heat map of the enzymatic activity of fungi, eubacteria and actinomycetes by sample type (blue represents the lower activity whereas red represents higher activity on the assay). Numbers correspond to sample number. RBBR: Lignin peroxidase activity in MEA-RBBR. CMC: Cellulase activity in CMC agar. LAC: Laccase activity on ABTS test. G: Glucanase activity. X: Xylanase activity. C: Cellobiase activity.

With 207 microbial isolates obtained from Passalid beetle gut samples (both from larvae and adults), including 88 fungi, 74 eubacteria and 44 actinomycetes in hand (Figure 4), we next tested their ability to break down plant cell wall biopolymers. In total, 68 isolates showed lignin peroxidation activity, including 33 fungi (37.5%), 20 eubacteria (26.7%) and 15 actinomycetes (34%), while 74 isolates had cellulolytic activity, including 30 fungi (34%), 17 eubacteria (22.7%) and 27 actinomycetes (61.3%). Only 23 isolates presented both activities (11 fungi, 6 eubacteria and 7 actinomycetes). Lignin peroxidation activity was significantly higher in fungi and actinomycetes, while cellulolytic activity was observed more frequently in actinomycetes than fungi. In both assays eubacteria showed significantly less activity (p<0.05) than the two other groups. Laccase activity was exclusively detected in fungi and was observed only in 10 isolates (11.3%).

The group of 23 isolates that exhibited positive activity on two or more assays on the screening phase was then subjected to further analysis for cellobiase, xylanase and glucanase activities (Table 2). Eight isolates presented the latter three activities, including the fungal strains 4505L (*Anthostomella* sp.), 4506K (*Hypocrea* sp.) and 4504W (*Cladophialophora* sp). There was also positive activity detected in the eubacteria 4504F (*Bacillus* sp.) and 4505D (*Pseudomonas* sp.) and the actinomycetes 4506AD, 4512 L and 4516K (all *Streptomyces* sp.). Cellobiase activity was observed only in 4505L (*Anthostomella* sp.) and in the eubacteria 4504F (*Bacillus* sp.) and 4505D (*Pseudomonas* sp.). *Hypomyces* sp. (4506K), *Hypocrea* sp. (4505I) and the Russulales fungal isolate 4509H also showed lacasse activity.

**Table 2:** Distribution of enzymatic activities of microbial isolates.

Isolate	Genus	RBBR^a^	CMC^b^	LAC^c^	G^d^	X^e^	C^f^	Total
4505L	*Anthostomella* sp.	2	2	0	2	0	4	10
4506AD	*Streptomyces* sp.	2	2	0	3	3	0	10
4512L	*Streptomyces* sp.	1	2	0	3	3	0	9
4506K	Hypocrea	2	1	1	2	2	0	8
4504F	*Bacillus* sp.	1	1	0	0	4	2	8
4505I	*Hypomyces* sp.	2	2	1	0	0	0	5
4504W	*Cladophialophora* sp.	2	1	0	2	0	0	5
4516K	*Streptomyces* sp.	1	2	0	0	2	0	5
4506P	*Metarhizium* sp.	2	2	0	0	0	0	4
4509H	*Fungi* *	1	2	1	0	0	0	4
4505D	*Pseudomonas* sp.	2	1	0	0	0	1	4
4503Q	Ascomycete	1	2	0	0	0	0	3
4504D	*Kluyvera* sp.	2	1	0	0	0	0	3
4505A	*Pseudomonas* sp.	2	1	0	0	0	0	3
4503T	*Streptomyces* sp.	1	2	0	0	0	0	3
4506AG	*Streptomyces* sp.	2	1	0	0	0	0	3
4503AB	*Streptomyces* sp.	1	2	0	0	0	0	3
4509J	*Chaetosphaeria* sp.	1	1	0	0	0	0	2
4507H	Ophiostomataceae	1	1	0	0	0	0	2
4507I	Ophiostomataceae	1	1	0	0	0	0	2
4515N	Ophiostomataceae	1	1	0	0	0	0	2
4512D	Enterobacteriacea	1	1	0	0	0	0	2
4505E	*Cupriavidus* sp.	1	1	0	0	0	0	2
4503S	*Bacteria* *	1	1	0	0	0	0	2

a Lignin peroxidase activity in MEA-RBBR.

b Cellulase activity in CMC agar.

c Laccase activity on ABTS test.

d Glucanase activity.

e Xylanase activity.

f Cellobiase activity.

*Taxonomic identification was not possible with the molecular markers available.

The fungal isolate 4505L (*Anthostomella*) and the actinomycete isolate 4506AD (*Streptomyces* sp.) had the highest scores both on the initial and the follow up assays, and thus showed the highest potential for the degradation of plant cell wall polymers (Table 2). These two highly active isolates were followed by another *Streptomyces* isolate (4512L), a *Hypocrea* isolate (4506K), and a *Bacillus* isolate (4504F). In general, *Streptomyces* was the taxonomic group that displayed higher cellulase, lignin peroxidase and xylanase activities. Isolates belonging to the bacterial genera *Pseudomonas* and *Burkholderia*, known for their high metabolic versatility, also showed important cellulolytic activity. The heat map in Figure 4 indicates that most of the beetle specimens and gallery material contained bacteria and fungi with positive activity for degrading lignin and cellulose, two of the more recalcitrant components in plant cell walls. Glucanase, xylanase and cellobiase activities were slightly more represented in larvae, followed by gallery material and adults, however, these differences were not significant.

## Discussion

In this study we sought to explore the diversity of the cultivable microbiota that colonize the gut of larvae and adults of Costa Rican wood-feeding beetles in order to improve our understanding of this complex niche and to obtain microbial isolates with the ability to breakdown cellulose. To accomplish our goals, we isolated fungi, eubacteria and actinomycetes from the intestinal contents of specimens collected in several environmentally protected areas of Costa Rica. Although this strategy has an inherent bias because a minimum percentage of microorganisms are able to grow on artificial media, it is useful for finding unique exo-enzyme producing microorganisms that can be readily adapted to biotechnological processes [2]. After describing the macro- and microscopic morphology of each isolate, we performed phylogenetic analyses using known genetic markers and tested the capacity of Passalid beetle isolates to degrade the major components of plant cell walls. Our results indicate that this particular microecosystem is highly diverse in terms of species abundance, phylogenetic composition and metabolic capabilities, despite the inherent bias in the culture-dependent approach used.

In total, we isolated 611 microorganisms including fungi and bacteria. We observed a great diversity of morphotypes among these strains, especially for actinomycetes and fungi, showing a variety of colony shapes and pigments. In general, the diversity and species richness of the beetle gut cultivable microbiota was not significantly different between the specimens collected from the different protected areas (with the exception of fewer fungi isolated from beetles collected at Carara National Park). Furthermore, no significant differences were observed in the number and diversity of microorganisms obtained from larvae and adults, instead, both life stages seem to share the most frequently isolated groups. These results may strengthen the hypothesis that some of the microorganisms that are consistently isolated may have a close relationship with their insect hosts [2]. Further, several of the isolates in the GenBank database (Table S2) that provided best hits to the microbes isolated from beetle's digestive tracts were related to insects as termites, crickets, ants, wasps, cockroaches and fruit flies. We also found hits to the gut of other animals as the zebrafish *Danio rerio* and the tammar wallaby *Macropus* [35], [36], [37], [38], [39]. Additional support for this hypothesis is provided by the dominant members of the gut bacterial community recovered in this study, Firmicutes and Proteobacteria, which are also described as common inhabitants in the guts of different insect groups [6], including longicorn beetles (Cerambicidae) [40] and Lepidopteran larvae such as Gypsy Moth (Erebidae) [41] and tropical Saturniid caterpillars [42]. Within the Proteobacteria, the genera *Serratia, Pseudomonas, Citrobacter* and *Burkholderia* were the most abundant, while the Firmicutes genus *Bacillus* was the most frequenly isolated within this group. There are several reports describing the enzymatic capacities of these bacteria, including their ability to degrade xylan, cellulose and phenolic components in lignin; therefore they could be releasing easily accesible nutrients to their insect host [43], [44], [45], [46], [47]. *Bacillus* are also recognized for their ability to produce endo glycosyl hydrolases involved in these processes [48]. However, it is important to recognize that some of the microorganisms isolated in this study may be transient microbiota travelling with the ingested wood material through the insects' digestive systems, as the majority of the “best hits” found in GenBank were related to soil, rhizosphere and other environmental isolates,

Even though we isolated 241 actinomycetes in this study, we cannot conclude whether they are dominant members of the gut cultivable microbiota due to the selective media employed for their isolation. However, we can certainly affirm that this type of microorganism can be recovered consistently from the wood-feeding beetle gut. The dominant genus isolated in this study was *Streptomyces* (86% of isolates), but despite the lack of genetic diversity shown in their 16S rDNA sequences, they did show higher phenotypic diversity reflected in several different colony morphologies. *Streptomyces* are well known for their capacity to degrade cellulose and lignin, both resistant to degradation by most microorganisms [49], [50], as well as for their capacity to produce an array of secondary metabolites [51], [52], both of these traits can be exploited from beetle gut microbiota and could be key factors for establishing dominant symbiotic associations with their insect host.

Fungi are also well known members of insect gut microbial communities, including beetles [11], [53]. In this study, we isolated filamentous fungi from 23 different families; however this observed diversity may be representing the intrinsic flora of the beetle gut, or transitory associants passing through the intestinal tract. The isolation the entomopathogenic genera *Cordyceps* and *Metarhizium* may be an example of these phenomena, because their presence could be a result of ingestion within the diet rather than an active infection inside the beetle gut. However, the existence of an ecological relationship between the host and any of these fungi or other microbial symbionts cannot be discarded, specially because several of these microbes are known for their ability to secrete carbohydrate-degrading enzymes, and thus they might confer an adaptative advantage to cope with a continuous flux of complex polymeric materials, allowing the host to gain biomass in a niche filled with recalcitrant materials [54], [55]. The predominant fungal genus detected in this study was *Trichoderma*, which together with *Nectria*, also detected in the beetle gut, have been characterized as a bioenergy relevant microbial genome [46]. In addition to its ability to produce a wide array of enzymes including cellulases and xylanases [56], [57], *Trichoderma* is also well known as a biocontrol agent, reducing fungal diseases in crops [58]. In general, most isolated fungi belonged to the order Hypocreales, including *Trichoderma* and the genera *Metarhizium*, *Metacordyceps* and *Paecilomyces*, among others; several isolates from these genera have been involved in relevant biotechnological applications in agriculture, industry, environmental remediation and medicine [59], [60]. However, additional functional experiments and coevolution analysis are required to establish whether there is an ecological relationship between these isolates and their host. Analyzing the metabolic capacities of these isolates can provide evidence of potential benefits to the insects.

To accomplish this goal, we decided to focus on isolates from Passalid beetles and test their ability to degrade plant cell walls by endogenous enzymes. We chose these insects because our preliminary results indicated a rich and diverse microbiota, and their unusual cooperative and care-giving behavior among Coleopterans of sharing a decomposing log with their brood from birth through adulthood [15], [61] implied the potential for the selection and lateral transmission of beneficial microbes. Consequently, we found several highly active isolates in our enzymatic assays. A fungal isolate of the genus *Anthostomella*, family Xylariaceae, had the overal highest score in these tests. Some species of *Anthostomella* have been described as plant pathogens but little information is available about possible ecological relationships with beetles and other insects. Besides *Anthostomella*, other members of the Hypocreales group also showed significant scores in the experiment. In addition, microorganisms of the genera *Streptomyces*, *Bacillus* and *Pseudomonas* presented positive activities on these assays. These results strengthen the hypothesis that Passalid beetles depend on their gut microbiota to break down cellulose and obtain energy, liberating nutrients in the intestinal environment that can benefit both the microbiota and the beetle itself.

The nutrient-acquisition and biomass-degrading capabilities of the microbiota associated with insects such as termites [5], [62] and leaf-cutting ants [63] can be employed in multiple biotechnological processes [4]. Here we show that the gut of Costa Rican wood-feeding beetles is also a source of cultivable microorganisms with potential biotechnological applications. Some of these microbes may be involved in symbiotic relationships with the beetle hosts, helping them to obtain energy from recalcitrant plant material. Further research based on culture-independent approaches will enable an improved understanding of the structure and composition of the beetle gut microbiota and its role in the host physiology and ecology.

## Supporting Information

Figure S1 **Distribution of isolates from the gut of Costa Rican wood-feeding beetles.** A: Distribution of the total number of isolates from all beetles collected according to their family. B: Percentage of isolates obtained by number of specimens sampled in each family (no significant differences were observed, p<0.05).
(TIF)

Table S1 Distribution of bacterial and fungal isolates according to their taxonomy.
(DOCX)

Table S2 Morphological description and Blast analysis of isolates.
(DOCX)
