# Supplementary figures and images for: Uncovering the Cultivable Microbial Diversity of Costa Rican Beetles and Its Ability to Break Down Plant Cell Wall Components
Source: PLoS One. 2014 Nov 20;9(11):e113303. doi: 10.1371/journal.pone.0113303 (PMC4239062; doi:10.1371/journal.pone.0113303)

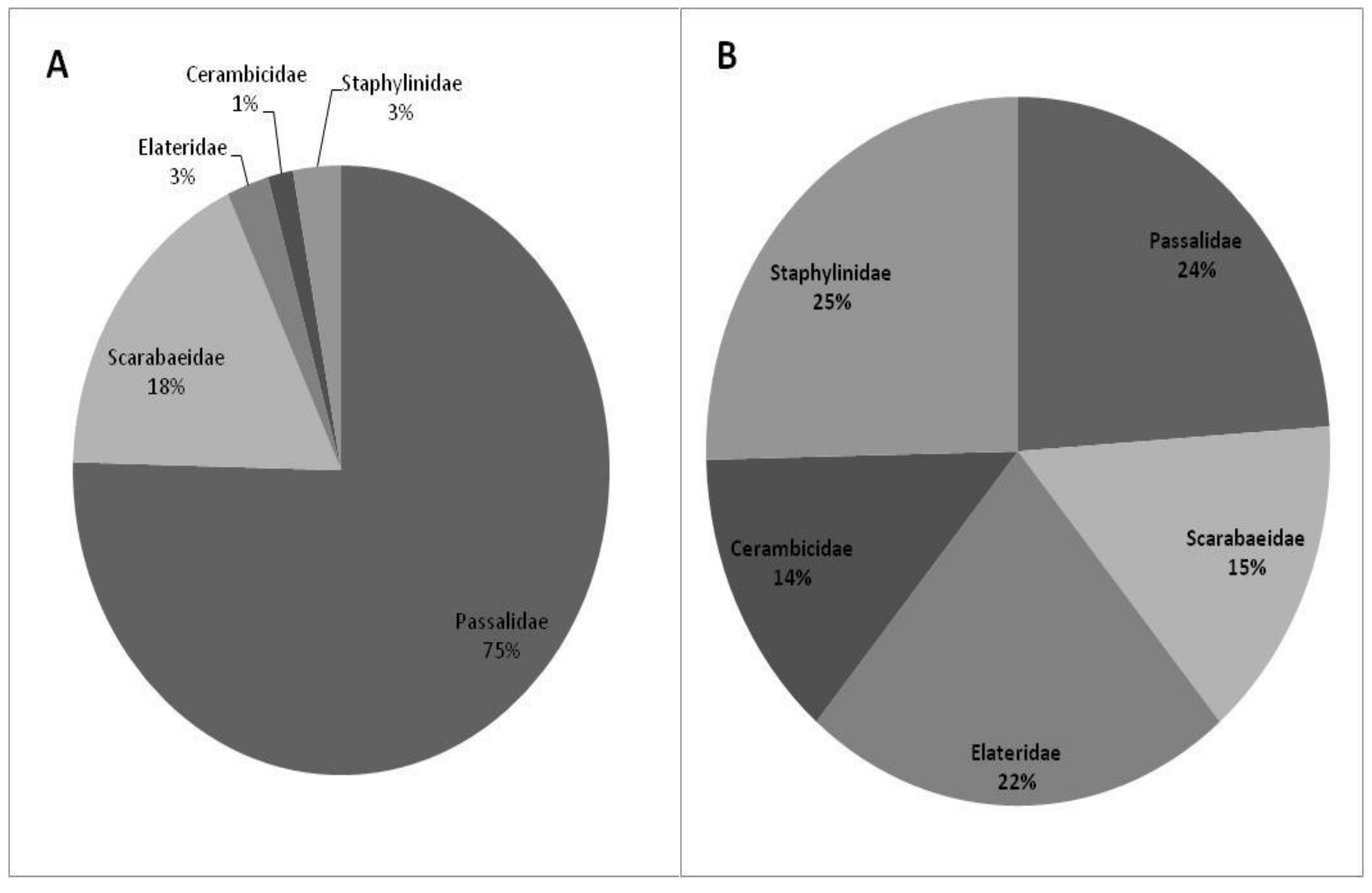

Supplement: Figure S1 — Distribution of isolates from the gut of Costa Rican wood-feeding beetles. A: Distribution of the total number of isolates from all beetles collected according to their family. B: Percentage of isolates obtained by number of specimens sampled in each family (no significant differences were observed, p<0.05). (TIF) [file pone.0113303.s001.tif]
