# Supplementary material for: Uncovering the Cultivable Microbial Diversity of Costa Rican Beetles and Its Ability to Break Down Plant Cell Wall Components
Source: PLoS One. 2014 Nov 20;9(11):e113303. doi: 10.1371/journal.pone.0113303 (PMC4239062; doi:10.1371/journal.pone.0113303)
Supplement: Table S1 — Distribution of bacterial and fungal isolates according to their taxonomy. (DOCX) [file pone.0113303.s002.docx]

Supplementary Table S1. Distribution of bacterial and fungal isolates according to their taxonomy.

| **KINGDOM** | **PHYLUM** | **ORDER** | **FAMILY** | **Number of isolates** |
| --- | --- | --- | --- | --- |
| Bacteria | Actinobacteria | Actinomycetales | Streptomycetaceae | 102 |
|  |  |  | Micrococcaceae | 4 |
|  |  |  | Mycobacteriaceae | 3 |
|  |  |  | Nocardiaceae | 1 |
|  |  |  | Corynebacteriaceae | 6 |
|  |  |  | Microbacteriaceae | 2 |
|  | Proteobacteria | Burkholderiales | Burkholderiaceae | 16 |
|  |  |  | Alcaligenaceae | 2 |
|  |  |  | Comamonadaceae | 1 |
|  |  | Enterobacteriales | Enterobacteriaceae | 31 |
|  |  | Pseudomonadales | Pseudomonadaceae | 4 |
|  |  | Neisseriales | Neisseriaceae | 3 |
|  |  | Rhizobiales | Rhizobiaceae | 1 |
|  |  | Vibrionales | Vibrionaceae | 1 |
|  |  | Xanthomonadales | Xanthomonadaceae | 4 |
|  | Firmicutes | Bacillales | Bacillaceae | 31 |
|  |  |  | Staphylococcaceae | 1 |
|  |  | Lactobacillales | Enterococcaceae | 14 |
|  |  |  | Streptococcaceae | 9 |
|  | No identified |  |  | 4 |
| Fungi | Ascomycota | Acarosporales | Acarosporaceae | 2 |
|  |  | Capnodiales | No identified | 2 |
|  |  | Chaetosphaeriales | Chaetosphaeriaceae | 11 |
|  |  | Chaetothyriales | Herpotrichiellaceae | 6 |
|  |  | Eurotiales | Trichocomaceae | 3 |
|  |  | Glomerellales | Plectosphaerellaceae | 5 |
|  |  | Hypocreales | Bionectriaceae | 5 |
|  |  | Hypocreales | Clavicipitaceae | 8 |
|  |  |  | Ophiocordycipitaceae | 7 |
|  |  |  | Nectriaceae | 10 |
|  |  |  | Hypocreaceae | 12 |
|  |  | Microascales | Microascaceae | 1 |
|  |  | Ophiostomatales | Ophiostomataceae | 14 |
|  |  | Pleosporales | Arthopyreniaceae | 2 |
|  |  |  | Phaeosphaeriaceae | 1 |
|  |  |  | Didymellaceae | 1 |
|  |  |  | Sporormiaceae | 1 |
|  |  | Saccharomycetales | Saccharomycetaceae | 1 |
|  |  | Xylariales | Xylariaceae | 1 |
|  |  |  | Amphisphaeriaceae | 1 |
|  |  | Xylariales | Diatrypaceae | 1 |
|  |  | Onygenales | Myxotrichaceae | 9 |
|  | Basidiomycota | Dacrymycetales | Dacrymycetaceae | 4 |
|  | Zygomycota | Mucorales | Mucoraceae | 4 |
|  | No identified |  |  | 25 |

* Sequence information was insufficient for taxonomic identification of the isolate.
