# Supplementary material for: Uncovering the Cultivable Microbial Diversity of Costa Rican Beetles and Its Ability to Break Down Plant Cell Wall Components
Source: PLoS One. 2014 Nov 20;9(11):e113303. doi: 10.1371/journal.pone.0113303 (PMC4239062; doi:10.1371/journal.pone.0113303)
Supplement: Table S2 — Morphological description and Blast analysis of isolates. (DOCX) [file pone.0113303.s003.docx]

Supplementary Table S2. Morphological description and Blast analysis of isolates

| **Isolation id** | **Host Family** | **Isolation media** | **Margin** | **Form** | **Elevation** | **Descripcion/Color** | **GenBank best hit** | **GenBank code of best hit** | **Sequence length** | **Coverage** | **evalue** | **Bitscore** | **%identification** | **Source of Blast best hit** |
| --- | --- | --- | --- | --- | --- | --- | --- | --- | --- | --- | --- | --- | --- | --- |
| INBio_4503AB | Passalidae | ISP2 media | Entire | Circular | Umbonate | 57 - Sulphur yellow | Streptomyces sp. GE 90852 | AJ519938 | 1421 | 99 | 0 | 2588 | 99.58 | n/a |
| INBio_4503K | Passalidae | ISP2 media | Undulated | Irregular | Umbonate | 223D - Tawny olive | Streptomyces niveoruber | AB741463 | 1421 | 99 | 0 | 2577 | 99.44 | Soil |
| INBio_4503T | Passalidae | ISP2 media | Filamentous | Circular | Umbonate | 92 - Pale horn | Streptomyces sp. GE 90852 | AJ519938 | 1420 | 99 | 0 | 2593 | 99.65 | n/a |
| INBio_4503U | Passalidae | ISP2 media | Filamentous | Circular | Convex | 54 - Cream | Streptomyces sp. CA2 | AB622247 | 1422 | 99 | 0 | 2595 | 99.65 | Termite gut |
| INBio_4503V | Passalidae | ISP2 media | Filamentous | Circular | Umbonate | 44 - Smoke gray | Streptomyces sp. GE 90852 | AJ519938 | 1420 | 100 | 0 | 2599 | 99.72 | n/a |
| INBio_4503X | Passalidae | ISP2 media | Entire | Circular | Umbonate | 92 - Pale horn | Streptomyces sp. CS392 | JN128646 | 1420 | 99 | 0 | 2584 | 99.51 | Soil |
| INBio_4503Y | Passalidae | ISP2 media | Filamentous | Circular | Umbonate | 54 - Cream | Streptomyces sp. MI264-NF2 | AB695175 | 1419 | 99 | 0 | 2545 | 99.01 | n/a |
| INBio_4503Z | Passalidae | ISP2 media | Filamentous | Circular | Umbonate | 223 - Raw umber | Streptomyces sp. TOHO-O682 | AB849957 | 1420 | 100 | 0 | 2579 | 99.44 | Soil |
| INBio_4504AA | Passalidae | ISP2 media | Entire | Circular | Umbonate | 44 - Smoke gray | Streptomyces sp. CS392 | JN128646 | 1246 | 99 | 0 | 2276 | 99.6 | Soil |
| INBio_4504AB | Passalidae | ISP2 media | Filamentous | Circular | Umbonate | 54 - Cream | Streptomyces sp. CA2 | AB622247 | 1423 | 99 | 0 | 2591 | 99.58 | Termite gut |
| INBio_4504AC | Passalidae | ISP2 media | Filamentous | Circular | Umbonate | 223C - Sayal brown | Streptomyces niveoruber | AB741463 | 1432 | 99 | 0 | 2514 | 98.53 | Soil |
| INBio_4504AE | Passalidae | ISP2 media | Entire | Circular | Umbonate | 223C - Sayal brown | Streptomyces sp. TM10 | JQ864373 | 1382 | 98 | 0 | 2494 | 99.28 | Gut *Reticulitermes chinensis Snyder* |
| INBio_4504AF | Passalidae | ISP2 media | Entire | Circular | Umbonate | 223D - Tawny olive | Streptomyces sp. CS392 | JN128646 | 1422 | 99 | 0 | 2580 | 99.44 | Soil |
| INBio_4504AG | Passalidae | ISP2 media | Filamentous | Circular | Umbonate | 56 - Straw yellow | Streptomyces sp. GE 90852 | AJ519938 | 1429 | 99 | 0 | 2553 | 99.02 | n/a |
| INBio_4504AI | Passalidae | ISP2 media | Filamentous | Circular | Umbonate | 54 - Cream | Streptomyces sp. CS392 | JN128646 | 1393 | 99 | 0 | 2540 | 99.57 | Soil |
| INBio_4504AO | Passalidae | ISP2 media | Filamentous | Circular | Umbonate | 219 - Sepia | Streptosporangium sp. TFS 1184 | EF216357 | 1426 | 99 | 0 | 2564 | 99.16 | Shallow water sediment |
| INBio_4504AQ | Passalidae | ISP2 media | Filamentous | Circular | Umbonate | 45 - Smoke gray | Streptomyces sp. CRCB1 | JN617221 | 1395 | 99 | 0 | 2462 | 98.57 | Coffee residue compost |
| INBio_4504AR | Passalidae | ISP2 media | Filamentous | Circular | Umbonate | 124 - Buff | Streptomyces niveoruber | AB741463 | 1412 | 99 | 0 | 2584 | 99.72 | Soil |
| INBio_4504B | Passalidae | ISP2 media | Filamentous | Circular | Umbonate | 124 - Buff | Streptomyces ginsengisoli | AB245393 | 1440 | 100 | 0 | 2597 | 99.24 | Soil |
| INBio_4504C | Passalidae | ISP2 media | n/a | n/a | n/a | n/a | Microbacterium sp. bE6(2011) | JF772479 | 1375 | 99 | 0 | 2353 | 97.6 | Soil |
| INBio_4504E | Passalidae | ISP2 media | n/a | n/a | n/a | n/a | Microbacterium sp. bE6(2011) | JF772479 | 1423 | 100 | 0 | 2431 | 97.61 | Soil |
| INBio_4504G | Passalidae | ISP2 media | n/a | n/a | n/a | n/a | Microbacterium takaoensis | AB201047 | 1416 | 99 | 0 | 2543 | 99.08 | n/a |
| INBio_4504O | Passalidae | ISP2 media | Filamentous | Circular | Convex | 54 - Cream | Streptomyces misawanensis | AB184533 | 1414 | 99 | 0 | 2577 | 99.58 | n/a |
| INBio_4504P | Passalidae | ISP2 media | Filamentous | Circular | Convex | 219D - Beige | Streptomyces sp. GE 90852 | AJ519938 | 1411 | 99 | 0 | 2590 | 99.79 | n/a |
| INBio_4504X | Passalidae | ISP2 media | Filamentous | Circular | Umbonate | 92 - Pale horn | Streptomyces microflavus | AB045861 | 1405 | 99 | 0 | 2547 | 99.43 | n/a |
| INBio_4504Y | Passalidae | ISP2 media | Entire | Circular | Umbonate | 44 - Smoke gray | Streptomyces sp. GE 90852 | AJ519938 | 1416 | 99 | 0 | 2591 | 99.72 | n/a |
| INBio_4504Z | Passalidae | ISP2 media | Entire | Circular | Umbonate | 92 - Pale horn | Streptomyces sp. MI264-NF2 | AB695175 | 1420 | 99 | 0 | 2553 | 99.08 | n/a |
| INBio_4505B | Substrate related to Passalidae | ISP2 media | n/a | n/a | n/a | n/a | Microbacterium sp. bE6(2011) | JF772479 | 1433 | 99 | 0 | 2438 | 97.49 | Soil |
| INBio_4505P | Substrate related to Passalidae | ISP2 media | Filamentous | Circular | Umbonate | 121D - Pale pinkish buf | Streptomyces sp. SB-B41 | DQ904544 | 1390 | 99 | 0 | 2529 | 99.5 | n/a |
| INBio_4505Q | Substrate related to Passalidae | ISP2 media | Filamentous | Circular | Umbonate | 219D - Beige | Streptomyces sp. CS392 | JN128646 | 1236 | 100 | 0 | 2263 | 99.68 | Soil |
| INBio_4505T | Substrate related to Passalidae | ISP2 media | Filamentous | Filamentous | Umbonate | 54 - Cream | Streptomyces sp. CA2 | AB622247 | 1354 | 99 | 0 | 2444 | 99.26 | Termite gut |
| INBio_4505U | Substrate related to Passalidae | ISP2 media | Erose | Circular | Umbonate | 8 - Carmine | Streptomyces sp. 3446 | DQ673872 | 1425 | 99 | 0 | 2577 | 99.3 | n/a |
| INBio_4506AC | Passalidae | ISP2 media | Filamentous | Circular | Umbonate | 221D - Light russet vin | Streptomyces sp. WPCB077 | FJ006885 | 1417 | 99 | 0 | 2582 | 99.58 | Freshwater of Woopo wetland |
| INBio_4506AD | Passalidae | ISP2 media | Filamentous | Circular | Umbonate | 106 - Salmon | Streptomyces griseoruber | HQ335354 | 1440 | 99 | 0 | 2638 | 99.72 | n/a |
| INBio_4506AE | Passalidae | ISP2 media | Filamentous | Circular | Umbonate | 124 - Buff | Streptomyces angustmyceticus | AB184817 | 1423 | 99 | 0 | 2608 | 99.79 | n/a |
| INBio_4506AG | Passalidae | ISP2 media | Filamentous | Circular | Umbonate | 53 - Buff-yellow | Streptomyces sp. SB-25 | DQ994688 | 1416 | 99 | 0 | 2608 | 99.93 | n/a |
| INBio_4506AJ | Passalidae | ISP2 media | Entire | Circular | Umbonate | 123C - Yellow ocher | Streptomyces sp. O10 | HG428670 | 1440 | 99 | 0 | 2549 | 98.68 | Oubeira Lake |
| INBio_4506AL | Passalidae | ISP2 media | Filamentous | Circular | Flat | 6 - Salmon | Streptomyces acidiscabies | FJ546739 | 1430 | 100 | 0 | 2601 | 99.51 | n/a |
| INBio_4506AM | Passalidae | ISP2 media | Filamentous | Circular | Umbonate | 124 - Buff | Streptomyces sp. GB21 | JX965400 | 1408 | 99 | 0 | 2518 | 99.01 | Soil |
| INBio_4506AN | Passalidae | ISP2 media | Filamentous | Circular | Umbonate | 42 - Olive-gray | Streptomyces mirabilis | FJ481081 | 1380 | 100 | 0 | 2542 | 99.93 | Soil |
| INBio_4506AO | Passalidae | ISP2 media | Entire | Circular | Convex | 54 - Cream | Rhodococcus sp. TMP2 | HE578013 | 1410 | 99 | 0 | 2483 | 98.44 | n/a |
| INBio_4506AP | Passalidae | ISP2 media | Filamentous | Circular | Umbonate | 42 - Olive-gray | Streptomyces sp. BK170 | FR692108 | 1432 | 99 | 0 | 2617 | 99.65 | Soil |
| INBio_4506U | Passalidae | ISP2 media | Filamentous | Circular | Raised | 124 - Buff | Streptomyces sp. HW24 | KF194340 | 1420 | 99 | 0 | 2536 | 98.94 | Larvae feces Japanese rhinoceros beetle |
| INBio_4506V | Passalidae | ISP2 media | Entire | Circular | Raised | 223D - Tawny olive | Streptomyces sp. CS392 | JN128646 | 1404 | 99 | 0 | 2580 | 99.86 | Soil |
| INBio_4506W | Passalidae | ISP2 media | Filamentous | Circular | Umbonate | 124 - Buff | Streptomyces sp. NT 0401 | DQ985808 | 1418 | 100 | 0 | 2551 | 99.15 | n/a |
| INBio_4506X | Passalidae | ISP2 media | Entire | Circular | Raised | 53 - Buff-yellow | Streptacidiphilus sp. Aac-103 | AB180760 | 1413 | 99 | 0 | 2462 | 98.16 | Soil |
| INBio_4506Y | Passalidae | ISP2 media | Filamentous | Circular | Umbonate | 119D - Drab-gray | Streptomyces sp. KS32 | AB373982 | 1409 | 99 | 0 | 2584 | 99.79 | Soil |
| INBio_4506Z | Passalidae | ISP2 media | Filamentous | Circular | Umbonate | 219D - Beige | Streptomyces sp. ACT-0092 | GQ924532 | 1424 | 99 | 0 | 2514 | 98.6 | Root *Tristiropsis acutangula* |
| INBio_4507D | Passalidae | ISP2 media | n/a | n/a | n/a | n/a | Microbacterium takaoensis | AB201047 | 1413 | 99 | 0 | 2564 | 99.43 | n/a |
| INBio_4507J | Passalidae | ISP2 media | Filamentous | Circular | Flat | 223D - Tawny olive | Streptomyces sp. MI264-NF2 | KC554772 | 1421 | 99 | 0 | 2560 | 99.16 | n/a |
| INBio_4507K | Passalidae | ISP2 media | Erose | Circular | Umbonate | 123B - Clay | Streptomyces sp. CS392 | JN128646 | 1419 | 99 | 0 | 2575 | 99.44 | Soil |
| INBio_4507L | Passalidae | ISP2 media | Entire | Circular | Umbonate | 121D - Pale pinkish buf | Microbacterium sp. BD-16 | GU085224 | 1390 | 99 | 0 | 2435 | 98.35 | Soil |
| INBio_4507M | Passalidae | ISP2 media | Filamentous | Circular | Umbonate | 56 - Straw yellow | Rhodococcus sp. TMP2 | GU444067 | 1425 | 99 | 0 | 2484 | 98.18 | n/a |
| INBio_4507O | Passalidae | ISP2 media | Entire | Circular | Convex | 92 - Pale horn | Streptomyces seoulensis | EU595010 | 1393 | 99 | 0 | 2483 | 98.85 | Soil |
| INBio_4508G | Substrate related to Passalidae | ISP2 media | Filamentous | Circular | Umbonate | 124 - Buff | Streptomyces sp. CS392 | JN128646 | 1419 | 99 | 0 | 2608 | 99.86 | Soil |
| INBio_4508H | Substrate related to Passalidae | ISP2 media | Filamentous | Circular | Convex | 219C - Cinnamon-drab | Streptomyces sp. JR-4;Streptomyces sp. JR-43 | HM748597 | 1409 | 100 | 0 | 2573 | 99.65 | Rhizosphere soil |
| INBio_4508I | Substrate related to Passalidae | ISP2 media | Filamentous | Filamentous | Umbonate | 53 - Buff-yellow | Streptomyces niveoruber | AB741463 | 1414 | 99 | 0 | 2582 | 99.65 | Soil |
| INBio_4509N | Passalidae | ISP2 media | n/a | n/a | n/a | #N/A | Streptomyces nodosus | AF114036 | 1413 | 100 | 0 | 2580 | 99.65 | n/a |
| INBio_4511F | Substrate related to Passalidae | ISP2 media | Entire | Circular | Pulvinada | 54 - Cream | Mycobacterium chelonae | NR_042918 | 1412 | 99 | 0 | 2595 | 99.86 | n/a |
| INBio_4511G | Substrate related to Passalidae | ISP2 media | Lobulado | Irregular | Flat | 54 - Cream | Microbacteriaceae bacterium RU-04 | HQ832502 | 1410 | 99 | 0 | 2499 | 98.72 | Insect gut (*Gryllotalpa africana*) |
| INBio_4512K | Passalidae | ISP2 media | Filamentous | Filamentous | Umbonate | 54 - Cream | Streptomyces sp. CA2 | AB622247 | 1393 | 100 | 0 | 2543 | 99.64 | Termite gut |
| INBio_4512L | Passalidae | ISP2 media | Filamentous | Circular | Umbonate | 41 - Ferruginous | Streptomyces microflavus | AB045861 | 1438 | 99 | 0 | 2601 | 99.37 | n/a |
| INBio_4512M | Passalidae | ISP2 media | Filamentous | Filamentous | Umbonate | 54 - Cream | Streptomyces sp. CA2 | AB622247 | 1415 | 100 | 0 | 2584 | 99.65 | Termite gut |
| INBio_4513M | Passalidae | ISP2 media | Entire | Circular | Umbonate | 223D - Tawny olive | Streptomyces ginsengisoli | FJ486369 | 1374 | 99 | 0 | 2470 | 99.2 | n/a |
| INBio_4513N | Passalidae | ISP2 media | Entire | Circular | Umbonate |  | Streptomyces ginsengisoli | FJ486369 | 1396 | 99 | 0 | 2542 | 99.57 | n/a |
| INBio_4513O | Passalidae | ISP2 media | Filamentous | Circular | Umbonate | 119D - Drab-gray | Streptomyces niveoruber | AB741463 | 1382 | 100 | 0 | 2529 | 99.71 | Soil |
| INBio_4513P | Passalidae | ISP2 media | Entire | Circular | Umbonate | 45 - Smoke gray | Streptomyces sp. KS32 | AB373982 | 1403 | 100 | 0 | 2573 | 99.79 | Soil |
| INBio_4513R | Passalidae | ISP2 media | Erose | Irregular | Flat | 124 - Buff | Mycobacterium sp. JAN2 | DQ866775 | 1424 | 99 | 0 | 2521 | 98.67 | Host=*Danio rerio* (zebrafish) |
| INBio_4513S | Passalidae | ISP2 media | Filamentous | Filamentous | Umbonate | 124 - Buff | Streptomyces niveoruber | AB741463 | 1430 | 99 | 0 | 2586 | 99.37 | Soil |
| INBio_4513T | Passalidae | ISP2 media | Filamentous | Circular | Pulvinada | 121D - Pale pinkish buf | Streptomyces ginsengisoli | FJ486369 | 1387 | 99 | 0 | 2531 | 99.64 | n/a |
| INBio_4513U | Passalidae | ISP2 media | n/a | n/a | n/a | #N/A | Streptomyces atratus | KF475815 | 1435 | 100 | 0 | 2632 | 99.79 | Tea roots |
| INBio_4515O | Passalidae | ISP2 media | Filamentous | Circular | Umbonate | 124 - Buff | Streptomyces uncialis | JN177509 | 1413 | 99 | 0 | 2510 | 98.8 | n/a |
| INBio_4515P | Passalidae | ISP2 media | Filamentous | Filamentous | Convex | 121D - Pale pinkish buf | Streptomyces uncialis | JN177509 | 1415 | 99 | 0 | 2514 | 98.8 | n/a |
| INBio_4515Q | Passalidae | ISP2 media | Filamentous | Filamentous | Umbonate | 223D - Tawny olive | Streptomyces sp. O10 | HG428670 | 1432 | 99 | 0 | 2569 | 99.09 | Oubeira Lake |
| INBio_4516AA | Passalidae | ISP2 media | n/a | n/a | n/a | #N/A | Streptomyces sp. SXY83 | GU045536 | 1429 | 99 | 0 | 2551 | 98.88 | Soil |
| INBio_4516AB | Passalidae | ISP2 media | Filamentous | Circular | Umbonate | 6 - Salmon | Streptomyces sp. 172124 | HQ992718 | 1425 | 99 | 0 | 2621 | 99.86 | n/a |
| INBio_4516K | Passalidae | ISP2 media | Filamentous | Circular | Umbonate | 119D - Drab-gray | Streptomyces sp. GE 90852 | AJ519938 | 1398 | 99 | 0 | 2566 | 99.79 | n/a |
| INBio_4516L | Passalidae | ISP2 media | Entire | Circular | Flat | 223D - Tawny olive | Streptomyces sp. BK170 | FR692108 | 1406 | 100 | 0 | 2555 | 99.5 | Soil |
| INBio_4516M | Passalidae | ISP2 media | Filamentous | Circular | Umbonate | 121D - Pale pinkish buf | Streptomyces sp. GE 90852 | AJ519938 | 1388 | 100 | 0 | 2527 | 99.57 | n/a |
| INBio_4516N | Passalidae | ISP2 media | Filamentous | Circular | Umbonate | 92 - Pale horn | Streptomyces sp. KS32 | AB373982 | 1398 | 99 | 0 | 2540 | 99.5 | Soil |
| INBio_4516P | Passalidae | ISP2 media | Filamentous | Filamentous | Convex | 121D - Pale pinkish buf | Rhodococcus sp. PLL-1 | EU127452 | 1402 | 99 | 0 | 2477 | 98.57 | n/a |
| INBio_4516Q | Passalidae | ISP2 media | Entire | Circular | Raised | 121D - Pale pinkish buf | Streptomyces sp. CS392 | JN128646 | 1418 | 99 | 0 | 2612 | 99.93 | Soil |
| INBio_4516S | Passalidae | ISP2 media | Filamentous | Circular | Umbonate | 124 - Buff | Streptomyces sp. ACT-0092 | GQ924532 | 1370 | 99 | 0 | 2468 | 99.27 | Root *Tristiropsis acutangula* |
| INBio_4516T | Passalidae | ISP2 media | Filamentous | Filamentous | Umbonate | 54 - Cream | Streptomyces purpureus | NR_042292 | 1392 | 99 | 0 | 2540 | 99.64 | n/a |
| INBio_4516U | Passalidae | ISP2 media | Filamentous | Circular | Umbonate | 124 - Buff | Streptomyces sp. BK170 | FR692108 | 1404 | 100 | 0 | 2558 | 99.57 | Soil |
| INBio_4516V | Passalidae | ISP2 media | Filamentous | Circular | Umbonate | 53 - Buff-yellow | Streptomyces sp. S-2-4 | JN866746 | 1371 | 100 | 0 | 2492 | 99.49 | Soil |
| INBio_4516W | Passalidae | ISP2 media | Filamentous | Circular | Umbonate | 124 - Buff | Streptomyces sp. BK170 | FR692108 | 1429 | 99 | 0 | 2604 | 99.58 | Soil |
| INBio_4516X | Passalidae | ISP2 media | Filamentous | Circular | Umbonate | 124 - Buff | Streptomyces sp. A00103 | EF690255 | 1405 | 99 | 0 | 2525 | 99.22 | Twig of Taxus |
| INBio_4516Y | Passalidae | ISP2 media | Filamentous | Circular | Umbonate | 121D - Pale pinkish buf | Streptomyces sp. ACT-0092 | GQ924532 | 1379 | 100 | 0 | 2470 | 99.13 | Root *Tristiropsis acutangula* |
| INBio_4516Z | Passalidae | ISP2 media | Filamentous | Circular | Umbonate | 132D - Flesh ocher | Streptomyces microflavus | AB045861 | 1424 | 99 | 0 | 2601 | 99.65 | n/a |
| INBio_4517F | Substrate related to Passalidae | ISP2 media | Filamentous | Circular | Umbonate | 124 - Buff | Streptomyces sp. BK170 | FR692108 | 1379 | 100 | 0 | 2525 | 99.71 | Soil |
| INBio_4517H | Substrate related to Passalidae | ISP2 media | Filamentous | Circular | Umbonate | 124 - Buff | Streptomyces bungoensis | JN180215 | 1379 | 99 | 0 | 2501 | 99.42 | Soil |
| INBio_4517J | Substrate related to Passalidae | ISP2 media | Filamentous | Circular | Umbonate | 124 - Buff | Streptomyces ginsengisoli | AB245393 | 1431 | 99 | 0 | 2580 | 99.23 | Soil |
| INBio_4517K | Substrate related to Passalidae | ISP2 media | Filamentous | Circular | Convex | 221D - Light russet vin | Streptomyces sp. 3446 | DQ673872 | 1423 | 100 | 0 | 2603 | 99.72 | n/a |
| INBio_4517M | Substrate related to Passalidae | ISP2 media | Filamentous | Circular | Convex | 121D - Pale pinkish buf | Streptomyces niveoruber | AB741463 | 1419 | 99 | 0 | 2586 | 99.58 | Soil |
| INBio_4036H | Passalidae | LB media | Undulate | Irregular | Raised | 92 - Pale horn | Enterobacter sp. GfU-1 | AB673457 | 1403 | 100 | 0 | 2571 | 99.71 | Gut of a wood-feeding. Host=*Glyptotermes fuscus* |
| INBio_4037H | Passalidae | LB media | Undulate | Irregular | Convex | 92 - Pale horn | uncultured Achromobacter sp. | GQ416596 | 1396 | 100 | 0 | 2555 | 99.71 | Biological degreasing systems |
| INBio_4037I | Passalidae | LB media | Entire | Circular | Convex | 92 - Pale horn | Kluyvera cryocrescens | AM992189 | 1403 | 100 | 0 | 2591 | 100 | Spring water |
| INBio_4038F | Passalidae | LB media | Entire | Circular | Convex | 92 - Pale horn | Klebsiella variicola | JX968498 | 1403 | 100 | 0 | 2591 | 100 | Barley malt |
| INBio_4038I | Passalidae | LB media | Entire | Circular | Convex | 92 - Pale horn | Lactococcus garvieae | JQ795812 | 1368 | 100 | 0 | 2492 | 99.56 | Roach gut content. Host=Rutilus rutilus. |
| INBio_4038J | Passalidae | LB media | Erose | Circular | Convex | 92 - Pale horn | Cupriavidus pauculus | AM418462 | 1395 | 100 | 0 | 2555 | 99.71 | Soil |
| INBio_4039AB | Passalidae | LB media | Filamentous | Rhizoid | Raised | 92 - Pale horn | Bacillaceae bacterium KVD-1790-04 | DQ490403 | 1414 | 100 | 0 | 2606 | 99.93 | Volcanic deposits |
| INBio_4039AD | Passalidae | LB media | Undulate | Irregular | Raised | 92 - Pale horn | Bacillus thuringiensis | KF641792 | 1374 | 100 | 0 | 2531 | 99.93 | Rumen. Host=camel |
| INBio_4039AG | Passalidae | LB media | Filamentous | Filamentous | Flat | 92 - Pale horn | Bacillus sp. NT4 | GU458276 | 1154 | 100 | 0 | 2076 | 99.22 | Termite gut. Host=Reticulitermes speratus |
| INBio_4039P | Passalidae | LB media | Filamentous | Rhizoid | Umbonate | 92 - Pale horn | Bacillus thuringiensis YBT-1518 | CP005935 | 1415 | 100 | 0 | 2595 | 99.79 | Soil |
| INBio_4039T | Passalidae | LB media | Filamentous | Rhizoid | Umbonate | 92 - Pale horn | Bacillus subtilis | KC414931 | 1361 | 100 | 0 | 2514 | 100 | n/a |
| INBio_4039Y | Passalidae | LB media | Filamentous | Filamentous | Flat | 92 - Pale horn | Bacillus sp. SG10 | JX402425 | 1411 | 100 | 0 | 2575 | 99.65 | Sacred grove soil |
| INBio_4041I | Tenebrionidae | LB media | Entire | Circular | Convex | 92 - Pale horn | uncultured bacterium (Serratia) | DQ068899 | 1405 | 100 | 0 | 2595 | 100 | GI tract sample. Host=*Myrmeleon mobilis* |
| INBio_4042AS | Passalidae | LB media | Lobate | Irregular | Convex | 92 - Pale horn | Bacillus thuringiensis YBT-1518 | CP005935 | 1414 | 100 | 0 | 2612 | 100 | Soil |
| INBio_4042AY | Passalidae | LB media | Undulate | Irregular | Convex | 157 - Sulfur Yellow | Pseudomonas putida | AY622320 | 1400 | 100 | 0 | 2569 | 99.79 | n/a |
| INBio_4042AZ | Passalidae | LB media | Undulate | Irregular | Convex | 92 - Pale horn | Pseudomonas putida | AY622320 | 1400 | 100 | 0 | 2569 | 99.79 | n/a |
| INBio_4042AZ2 | Passalidae | LB media | Undulate | Irregular | Convex | 92 - Pale horn | Pseudomonas putida | AY622320 | 1401 | 100 | 0 | 2564 | 99.71 | n/a |
| INBio_4042BA | Passalidae | LB media | Entire | Circular | Convex | 92 - Pale horn | Achromobacter sp. JA81 | JN836430 | 1345 | 100 | 0 | 2464 | 99.7 | Soybean field |
| INBio_4046E | Passalidae | LB media | Entire | Circular | Convex | 92 - Pale horn | Citrobacter farmeri | JX393004 | 1334 | 100 | 0 | 2403 | 99.18 | Edible frog intestine |
| INBio_4048I | Passalidae | LB media | Entire | Circular | Convex | 92 - Pale horn | Rahnella aquatilis | GU204974 | 1405 | 100 | 0 | 2457 | 98.22 | Breeding sites of *Anopheles* |
| INBio_4048M | Scarabaeidae | LB media | Erose | Irregular | Raised | 92 - Pale horn | uncultured Serratia sp. | GQ416444 | 1405 | 100 | 0 | 2590 | 99.93 | Biological degreasing systems |
| INBio_4049C |  | LB media | Entire | Circular | Convex | 271 - White | Achromobacter xylosoxidans | AB680773 | 1394 | 100 | 0 | 2558 | 99.78 | n/a |
| INBio_4051L | Scarabaeidae | LB media | Entire | Circular | Raised | 92 - Pale horn | Stenotrophomonas maltophilia | KF150491 | 1406 | 100 | 0 | 2586 | 99.86 | Potassic trachyte |
| INBio_4052G | Passalidae | LB media | Erose | Circular | Umbonate | 92 - Pale horn | Bacillus toyonensis BCT-7112 | CP006863 | 1406 | 100 | 0 | 2597 | 100 | Soil |
| INBio_4052O | Passalidae | LB media | Entire | Circular | Convex | 8 - Carmine | uncultured Serratia sp. | GQ416470 | 1405 | 100 | 0 | 2595 | 100 | Biological degreasing systems |
| INBio_4054E | Passalidae | LB media | Filamentous | Rhizoid | Umbonate | 92 - Pale horn | Bacillus cereus | KF500919 | 1382 | 100 | 0 | 2542 | 99.86 | Intestinal tract of shrimp. Host=*Litopenaeus vannamei* |
| INBio_4054J | Passalidae | LB media | Entire | Circular | Convex | 92 - Pale horn | uncultured bacterium | JQ769897 | 1408 | 100 | 0 | 2601 | 100 | Soil |
| INBio_4054K | Passalidae | LB media | Entire | Circular | Convex | 92 - Pale horn | Enterobacter sp. FP2 | KF623096 | 1352 | 100 | 0 | 2459 | 99.48 | Legs of ants. Host=*Pseudomyrmex ferrugineus* |
| INBio_4057G | Tenebrionidae | LB media | Filamentous | Rhizoid | Umbonate | 92 - Pale horn | Bacillus subtilis | KC414931 | 1338 | 100 | 0 | 2471 | 100 | n/a |
| INBio_4058S |  | LB media | Entire | Circular | Convex | 92 - Pale horn | Enterobacteriaceae bacterium JML | KC119218 | 1408 | 100 | 0 | 2412 | 97.66 | Freshwater creek downstream |
| INBio_4062F |  | LB media | Filamentous | Rhizoid | Umbonate | 92 - Pale horn | Bacillus thuringiensis YBT-1518 | CP005935 | 1415 | 100 | 0 | 2606 | 99.93 | Soil |
| INBio_4062P |  | LB media | Filamentous | Rhizoid | Raised | 39 - Cinnamon | Staphylococcus haemolyticus | KF543100 | 1363 | 100 | 0 | 2512 | 99.93 | Soil |
| INBio_4064B |  | LB media | Entire | Circular | Convex | 172 - True violet | Chromobacterium sp. BW1 | DQ985277 | 1401 | 100 | 0 | 2575 | 99.86 | Blackbird Wetland soil |
| INBio_4064I |  | LB media | Entire | Circular | Pulvinada | 92 - Pale horn | Burkholderia acidipaludis | AB537485 | 1394 | 100 | 0 | 2414 | 97.92 | n/a |
| INBio_4065K |  | LB media | Entire | Circular | Convex | 92 - Pale horn | Burkholderia acidipaludis | AB537485 | 1394 | 100 | 0 | 2414 | 97.92 | n/a |
| INBio_4065M |  | LB media | Entire | Circular | Convex | 92 - Pale horn | Burkholderia rinojensis | KF650996 | 1396 | 100 | 0 | 2556 | 99.71 | Soil |
| INBio_4067B |  | LB media | Entire | Circular | Convex | 92 - Pale horn | Pantoea agglomerans | EU304255 | 1343 | 100 | 0 | 2464 | 99.78 | Larval gut. Host=*Hepialus gonggaensis* |
| INBio_4098AD |  | LB media | Entire | Circular | Convex | 92 - Pale horn | Burkholderia nodosa | AM284971 | 1395 | 100 | 0 | 2446 | 98.28 | Root nodule. Host=*Mimosa scabrella* |
| INBio_4098AJ | Passalidae | LB media | Entire | Circular | Convex | 92 - Pale horn | Burkholderia nodosa | AM284971 | 1396 | 100 | 0 | 2451 | 98.35 | Root nodule. Host=*Mimosa scabrella* |
| INBio_4098AQ | Passalidae | LB media | Entire | Circular | Convex | 271 - White | Variovorax sp. 2C1-21 | HQ385754 | 1398 | 100 | 0 | 2483 | 98.71 | Sewage |
| INBio_4098Y | Passalidae | LB media | Entire | Circular | Convex | 92 - Pale horn | Burkholderia sprentiae WSM5005 | HF549035 | 1217 | 100 | 0 | 2215 | 99.51 | Root nodule |
| INBio_4098Z | Passalidae | LB media | Lobate | Irregular | Convex | 92 - Pale horn | uncultured Citrobacter sp. | JQ885541 | 1405 | 100 | 0 | 2562 | 99.57 | Host=*Bactrocera correcta* |
| INBio_4099AF | Passalidae | LB media | Entire | Circular | Convex | 92 - Pale horn | uncultured bacterium | HE589892 | 1351 | 100 | 0 | 2226 | 96.52 | Rice phyllosphere |
| INBio_4099AG | Passalidae | LB media | Entire | Circular | Convex | 92 - Pale horn | Burkholderia sp. 59-VN4-1W | AB299575 | 1396 | 100 | 0 | 2473 | 98.64 | Soil |
| INBio_4099V | Passalidae | LB media | Entire | Circular | Convex | 92 - Pale horn | Citrobacter sp. TSB4 | JQ864380 | 1322 | 100 | 0 | 2420 | 99.7 | Gut. Host=*Reticulitermes chinensis* Snyder |
| INBio_4100H | Tenebrionidae | LB media | Entire | Circular | Convex | 92 - Pale horn | Burkholderia sp. Ms116 | FJ528268 | 1377 | 99 | 0 | 2409 | 98.26 | Legume nodules |
| INBio_4100K | Tenebrionidae | LB media | Entire | Circular | Convex | 92 - Pale horn | uncultured bacterium | DQ068868 | 1403 | 100 | 0 | 2591 | 100 | Host=*Myrmeleon mobilis* |
| INBio_4100L | Tenebrionidae | LB media | Entire | Circular | Convex | 75 - Mauve | Chromobacterium sp. BW1 | DQ985277 | 1401 | 100 | 0 | 2569 | 99.79 | Blackbird Wetland soil |
| INBio_4100Q | Tenebrionidae | LB media | Entire | Circular | Convex | 92 - Pale horn | Burkholderia tuberum | JX011000 | 1222 | 99 | 0 | 2185 | 98.94 | Biotite mineral and soil |
| INBio_4108C | Tenebrionidae | LB media | Undulate | Irregular | Convex | 92 - Pale horn | Bacillus thuringiensis YBT-1518 | CP005935 | 1414 | 100 | 0 | 2612 | 100 | /isolation_source="soil" |
| INBio_4108D | Tenebrionidae | LB media | Undulate | Irregular | Convex | 92 - Pale horn | Bacillus thuringiensis YBT-1518 | CP005935 | 1414 | 100 | 0 | 2597 | 99.79 | Soil |
| INBio_4108H | Tenebrionidae | LB media | Entire | Circular | Convex | 92 - Pale horn | Burkholderia sp. enrichment culture clone M18_07b_B | GQ306174 | 1347 | 99 | 0 | 2403 | 98.89 | Host=Camponotus cylindricus |
| INBio_4108J | Passalidae | LB media | Filamentous | Rhizoid | Convex | 92 - Pale horn | uncultured Citrobacter sp. | JQ885541 | 1360 | 100 | 0 | 2423 | 98.9 | Host=*Bactrocera correcta* |
| INBio_4109L | N/A | LB media | Filamentous | Irregular | Convex | 92 - Pale horn | Bacillus thuringiensis MC28 | CP003687 | 1414 | 100 | 0 | 2606 | 99.93 | n/a |
| INBio_4109M | N/A | LB media | Filamentous | Rhizoid | Raised | 92 - Pale horn | Bacillus thuringiensis YBT-1518 | CP005935 | 1415 | 100 | 0 | 2606 | 99.93 | Soil |
| INBio_4312S | Passalidae | LB media | Erose | n/a | n/a | n/a | Bacillus thuringiensis YBT-1518 | CP005935 | 1414 | 100 | 0 | 2612 | 100 | Soil |
| INBio_4358C | Passalidae | LB media | n/a | n/a | n/a | n/a | uncultured Serratia sp. | GQ416470 | 1405 | 100 | 0 | 2595 | 100 | Biological degreasing systems |
| INBio_4361C | Passalidae | LB media | n/a | n/a | n/a | n/a | uncultured Serratia sp. | GQ416470 | 1405 | 100 | 0 | 2595 | 100 | Biological degreasing systems |
| INBio_4362E | Passalidae | LB media | n/a | n/a | n/a | n/a | Serratia marcescens | AB680122 | 1405 | 100 | 0 | 2595 | 100 | n/a |
| INBio_4503A | Passalidae | LB media | n/a | n/a | n/a | n/a | Pseudomonas putida | JN982334 | 1400 | 100 | 0 | 2575 | 99.86 | Soil |
| INBio_4503C | Passalidae | LB media | n/a | n/a | n/a | n/a | Enterobacter sp. NCCP-240;Enterobacter sp. B58 | AB665214 | 1377 | 100 | 0 | 2519 | 99.71 | Mash bean (*Vigna mungo*) rhizosphere |
| INBio_4503D | Passalidae | LB media | n/a | n/a | n/a | n/a | Pseudomonas panipatensis | NR_044209 | 1391 | 99 | 0 | 2536 | 99.57 | Oil-contaminated site at Mathura Oil |
| INBio_4503E | Passalidae | LB media | n/a | n/a | n/a | n/a | Bacillus subtilis | KF475836 | 1404 | 100 | 0 | 2593 | 100 | Tea rhizosphere |
| INBio_4503G | Passalidae | LB media | Entire | Circular | Convex | 56 - Straw yellow | Bacillus sp. SG10 | JX402425 | 1415 | 100 | 0 | 2603 | 99.86 | Sacred grove soil |
| INBio_4504A | Passalidae | LB media | n/a | n/a | n/a | n/a | Enterobacter sp. S5-122 | JQ660160 | 1404 | 100 | 0 | 2558 | 99.57 | Plant tissue. Host=*Jatropha curcas* L. |
| INBio_4504D | Passalidae | LB media | n/a | n/a | n/a | n/a | Enterobacter sp. NCCP-240;Enterobacter sp. B58 | AB665214 | 1378 | 100 | 0 | 2518 | 99.64 | Mash bean (*Vigna mungo*) rhizosphere |
| INBio_4505A | Substrate related to Passalidae | LB media | n/a | n/a | n/a | n/a | Pseudomonas panipatensis | NR_044209 | 1391 | 99 | 0 | 2542 | 99.64 | Oil-contaminated site at Mathura Oil |
| INBio_4505E | Substrate related to Passalidae | LB media | n/a | n/a | n/a | n/a | Cupriavidus sp. KU-21 | AB266610 | 1395 | 100 | 0 | 2525 | 99.28 | n/a |
| INBio_4505H | Substrate related to Passalidae | LB media | n/a | n/a | n/a | n/a | Chromobacterium aquaticum | JQ582944 | 1401 | 100 | 0 | 2564 | 99.71 | Tivon waste stabilization pond |
| INBio_4506A | Passalidae | LB media | n/a | n/a | n/a | n/a | Lactococcus garvieae | JQ795812 | 1412 | 100 | 0 | 2603 | 99.93 | Roach gut content. Host=Rutilus rutilus |
| INBio_4506C | Passalidae | LB media | n/a | n/a | n/a | n/a | Bacillus sp. SG10 | JX402425 | 1415 | 100 | 0 | 2606 | 99.93 | Sacred grove soil |
| INBio_4506D | Passalidae | LB media | n/a | n/a | n/a | n/a | Bacillus thuringiensis YBT-1518 | CP005935 | 1414 | 100 | 0 | 2612 | 100 | Soil |
| INBio_4506E | Passalidae | LB media | n/a | n/a | n/a | n/a | Enterobacteriaceae bacterium strain FGI 57 | CP003938 | 1110 | 100 | 0 | 2017 | 99.46 | n/a |
| INBio_4506G | Passalidae | LB media | n/a | n/a | n/a | n/a | Lactococcus garvieae | JQ795812 | 1372 | 100 | 0 | 2529 | 99.93 | Roach gut content. Host=Rutilus rutilus. |
| INBio_4506I | Passalidae | LB media | n/a | n/a | n/a | n/a | Lysinibacillus fusiformis | KF475851 | 1415 | 100 | 0 | 2614 | 100 | Tea rhizosphere |
| INBio_4507A | Passalidae | LB media | n/a | n/a | n/a | n/a | uncultured bacterium | GQ358876 | 1421 | 99 | 0 | 2625 | 100 | Pouch young GIT. Host=*Macropus eugenii* |
| INBio_4507B | Passalidae | LB media | n/a | n/a | n/a | n/a | Enterobacteriaceae bacterium strain FGI 57 | CP003938 | 1406 | 100 | 0 | 2545 | 99.36 | n/a |
| INBio_4507C | Passalidae | LB media | n/a | n/a | n/a | n/a | Enterococcus gilvus | AB742448 | 1401 | 100 | 0 | 2556 | 99.64 | Raw cow milk |
| INBio_4508A | Substrate related to Passalidae | LB media | n/a | n/a | n/a | n/a | Burkholderia tropica | HQ023270 | 1396 | 100 | 0 | 2495 | 98.93 | Host=Saccharum hybrid cultivar CP 72-2086 |
| INBio_4508B | Substrate related to Passalidae | LB media | n/a | n/a | n/a | n/a | Dyella terrae | KF150459 | 1408 | 100 | 0 | 2579 | 99.72 | Potassic trachyte |
| INBio_4508C | Substrate related to Passalidae | LB media | n/a | n/a | n/a | n/a | Bacillus thuringiensis YBT-1518 | CP005935 | 1417 | 100 | 0 | 2597 | 99.79 | Soil |
| INBio_4508D | Substrate related to Passalidae | LB media | n/a | n/a | n/a | n/a | Burkholderia sp. hpud12.1 | AY691395 | 1371 | 100 | 0 | 2433 | 98.69 | Host=*Mimosa pudica* |
| INBio_4509A | Passalidae | LB media | n/a | n/a | n/a | n/a | Lactococcus lactis subsp. lactis KLDS 4.0325 | CP006766 | 1411 | 100 | 0 | 2599 | 99.93 | n/a |
| INBio_4509D | Passalidae | LB media | n/a | n/a | n/a | n/a | Enterobacteriaceae bacterium strain FGI 57 | CP003938 | 1405 | 100 | 0 | 2556 | 99.5 | n/a |
| INBio_4509E | Passalidae | LB media | n/a | n/a | n/a | n/a | Enterobacteriaceae bacterium JML | KC119218 | 1408 | 100 | 0 | 2412 | 97.66 | Freshwater creek downstream |
| INBio_4509F | Passalidae | LB media | n/a | n/a | n/a | n/a | Bacillus thuringiensis YBT-1518 | CP005935 | 1414 | 100 | 0 | 2612 | 100 | Soil |
| INBio_4510B | Passalidae | LB media | n/a | n/a | n/a | n/a | Enterobacteriaceae bacterium JML | KC119218 | 1408 | 100 | 0 | 2414 | 97.66 | Freshwater creek downstream |
| INBio_4510E | Passalidae | LB media | n/a | n/a | n/a | n/a | uncultured bacterium | GQ358876 | 1422 | 99 | 0 | 2614 | 99.86 | Pouch young GIT. Host=*Macropus eugenii* |
| INBio_4510F | Passalidae | LB media | n/a | n/a | n/a | n/a | Enterobacteriaceae bacterium JML | KC119218 | 1408 | 100 | 0 | 2418 | 97.73 | Freshwater creek downstream |
| INBio_4510G | Passalidae | LB media | n/a | n/a | n/a | n/a | Serratia rubidaea | AB680354 | 1408 | 100 | 0 | 2394 | 97.37 | n/a |
| INBio_4510K | Passalidae | LB media | Filamentous | Filamentous | Umbonate | 54 - Cream | Enterococcus gilvus | AB742448 | 1422 | 100 | 0 | 2615 | 99.86 | Raw cow milk |
| INBio_4511A | Substrate related to Passalidae | LB media | n/a | n/a | n/a | n/a | Burkholderia sp. CCGE1002 | CP002014 | 1396 | 100 | 0 | 2471 | 98.64 | n/a |
| INBio_4511B | Substrate related to Passalidae | LB media | n/a | n/a | n/a | n/a | Dyella terrae | KF150459 | 1408 | 100 | 0 | 2579 | 99.72 | Potassic trachyte |
| INBio_4511C | Substrate related to Passalidae | LB media | n/a | n/a | n/a | n/a | Dyella terrae | KF150459 | 1408 | 100 | 0 | 2573 | 99.64 | Potassic trachyte |
| INBio_4511D | Substrate related to Passalidae | LB media | n/a | n/a | n/a | n/a | Lysinibacillus sphaericus | KF527213 | 1222 | 100 | 0 | 2257 | 100 | Host=*Dendrobium officinale* |
| INBio_4512A | Passalidae | LB media | n/a | n/a | n/a | n/a | Enterobacteriaceae bacterium strain FGI 57 | CP003938 | 1366 | 100 | 0 | 2468 | 99.27 | n/a |
| INBio_4512B | Passalidae | LB media | n/a | n/a | n/a | n/a | Lactococcus garvieae | JQ795812 | 1412 | 100 | 0 | 2603 | 99.93 | Roach gut content. Host=Rutilus rutilus |
| INBio_4512C | Passalidae | LB media | n/a | n/a | n/a | n/a | Lactococcus garvieae | JQ795812 | 1412 | 100 | 0 | 2603 | 99.93 | Roach gut content. Host=Rutilus rutilus |
| INBio_4512D | Passalidae | LB media | n/a | n/a | n/a | n/a | Enterobacteriaceae bacterium strain FGI 57 | CP003938 | 1405 | 100 | 0 | 2540 | 99.29 | n/a |
| INBio_4512E | Passalidae | LB media | n/a | n/a | n/a | n/a | Lactococcus garvieae | JQ795812 | 1412 | 100 | 0 | 2603 | 99.93 | Roach gut content. Host=Rutilus rutilus |
| INBio_4513A | Passalidae | LB media | n/a | n/a | n/a | n/a | Enterobacteriaceae bacterium strain FGI 57 | CP003938 | 1405 | 100 | 0 | 2540 | 99.29 | n/a |
| INBio_4513D | Passalidae | LB media | n/a | n/a | n/a | n/a | Lactococcus lactis subsp. lactis KLDS 4.0325 | CP006766 | 1411 | 100 | 0 | 2599 | 99.93 | n/a |
| INBio_4513Q | Passalidae | LB media | Filamentous | Circular | Umbonate | 124 - Buff | Lactococcus lactis subsp. lactis KLDS 4.0325 | CP006766 | 1411 | 100 | 0 | 2593 | 99.86 | n/a |
| INBio_4514B | Substrate related to Passalidae | LB media | n/a | n/a | n/a | n/a | Lactococcus lactis subsp. lactis KLDS 4.0325 | CP006766 | 1414 | 100 | 0 | 2350 | 96.75 | n/a |
| INBio_4515A | Passalidae | LB media | n/a | n/a | n/a | n/a | uncultured Citrobacter sp. | JQ885541 | 1405 | 100 | 0 | 2562 | 99.57 | Host=*Bactrocera correcta* |
| INBio_4515B | Passalidae | LB media | n/a | n/a | n/a | n/a | Enterobacteriaceae bacterium SL4_G15 | HM235497 | 1405 | 100 | 0 | 2558 | 99.43 | Host=*Sirex noctilio* (larvae) |
| INBio_4515C | Passalidae | LB media | n/a | n/a | n/a | n/a | Lysinibacillus sphaericus | KF420486 | 1415 | 100 | 0 | 2608 | 99.93 | Waste water |
| INBio_4515E | Passalidae | LB media | n/a | n/a | n/a | n/a | uncultured Citrobacter sp. | JQ885541 | 1405 | 100 | 0 | 2562 | 99.57 | Host=*Bactrocera correcta* |
| INBio_4516A | Passalidae | LB media | n/a | n/a | n/a | n/a | Bacillus thuringiensis YBT-1518 | CP005935 | 1414 | 100 | 0 | 2612 | 100 | Soil |
| INBio_4516B | Passalidae | LB media | n/a | n/a | n/a | n/a | Enterobacteriaceae bacterium strain FGI 57 | CP003938 | 1406 | 100 | 0 | 2529 | 99.15 | n/a |
| INBio_4516D | Passalidae | LB media | n/a | n/a | n/a | n/a | Bacillus toyonensis BCT-7112 | CP006863 | 1397 | 100 | 0 | 2580 | 100 | Soil |
| INBio_4516E | Passalidae | LB media | n/a | n/a | n/a | n/a | Serratia sp. HUB-107 | FJ862037 | 1405 | 100 | 0 | 2595 | 100 | Phloem. Host=*Chamaecyparis lawsoniana* |
| INBio_4517C | Substrate related to Passalidae | LB media | n/a | n/a | n/a | n/a | Bacillus thuringiensis MC28 | CP003687 | 1415 | 100 | 0 | 2601 | 99.86 | n/a |
| INBio_4035B | Scarabaeidae | PDA agar | Filamentous | Filamentous | Raised | 271 - White | Trichoderma harzianum | KC330218 | 1221 | 100 | 0 | 2209 | 99.34 | Sugarcane bagasse waste decomposed |
| INBio_4039G | Passalidae | PDA agar | Erose | Circular | Convex | 146 - Leaf green | Talaromyces verruculosus | HM469420 | 1204 | 100 | 0 | 2183 | 99.42 | n/a |
| INBio_4065B |  | PDA agar | Erose | Circular | Convex | 271 - White | Trichoderma sp. 10 BRO-2013 | KF367524 | 561 | 99 | 0 | 1007 | 99.11 | Untreated drinking water sources |
| INBio_4065D |  | PDA agar | Entire | Circular | Convex | 271 - White | Isaria sp. KF0909_H3 | KC311491 | 613 | 100 | 0 | 1125 | 99.84 | n/a |
| INBio_4098N | Passalidae | PDA agar | Erose | Circular | Umbonate | 56 - Straw yellow | Trichoderma harzianum | JQ411365 | 639 | 100 | 0 | 1120 | 98.44 | Marine sponge |
| INBio_4503G | Passalidae | PDA agar | Entire | Circular | Convex | 56 - Straw yellow | Mucor sp. BESC196c | KC007161 | 953 | 76 | 0 | 1480 | 94.96 | Plant roots. Host=*Populus trichocarpa* |
| INBio_4503H | Passalidae | PDA agar | Filamentous | Circular | Raised | 271 - White | Elaphocordyceps ophioglossoides | AB027367 | 1217 | 100 | 0 | 2074 | 97.53 | n/a |
| INBio_4503I | Passalidae | PDA agar | Filamentous | Circular | Umbonate | 271 - White | Elaphocordyceps ophioglossoides | AB027367 | 1216 | 100 | 0 | 2078 | 97.62 | n/a |
| INBio_4503M | Passalidae | PDA agar | Erose | Circular | Umbonate | 271 - White | Dacrymyces chrysospermus | AF287855 | 852 | 71 | 0 | 1312 | 94.6 | n/a |
| INBio_4503N | Passalidae | PDA agar | Entire | Circular | Umbonate | 30 - Olive | Exophiala pisciphila | AF050272 | 1260 | 100 | 0 | 1917 | 94.44 | n/a |
| INBio_4503O | Passalidae | PDA agar | Filamentous | Circular | Umbonate | 92 - Pale horn | Eucasphaeria capensis | EF110619 | 1242 | 100 | 0 | 1834 | 93.64 | Host=*Eucalyptus* sp. |
| INBio_4503P | Passalidae | PDA agar | Erose | Circular | Umbonate | 92 - Pale horn | Dacrymyces chrysospermus | AF287855 | 851 | 71 | 0 | 1317 | 94.71 | n/a |
| INBio_4503Q | Passalidae | PDA agar | Entire | Circular | Umbonate | 271 - White | Umbilicaria esculenta | EU534208 | 1236 | 100 | 0 | 1434 | 88.11 | n/a |
| INBio_4504I | Passalidae | PDA agar | Filamentous | Circular | Plana | 92 - Pale horn | uncultured Ascomycota | EU490011 | 1220 | 100 | 0 | 1810 | 93.61 | Soil |
| INBio_4504J | Passalidae | PDA agar | Filamentous | Circular | Umbonate | 271 - White | Elaphocordyceps ophioglossoides | AB027367 | 1216 | 100 | 0 | 2078 | 97.62 | n/a |
| INBio_4504K | Passalidae | PDA agar | Filamentous | Irregular | Raised | 84 - Medium neutral gra | Chaetothyriales sp. M-Cre1-5 | HQ634635 | 1255 | 100 | 0 | 2185 | 98.17 | Host=*Crematogaster* sp. |
| INBio_4504L | Passalidae | PDA agar | Filamentous | Circular | Convex | 4 - Deep vinaceous | Neonectria punicea | HM534901 | 1219 | 100 | 0 | 1829 | 93.93 | Host=*Frangula alnus* |
| INBio_4504M | Passalidae | PDA agar | Filamentous | Circular | Convex | 271 - White | Elaphocordyceps ophioglossoides | AB027367 | 1224 | 100 | 0 | 2049 | 97.06 | n/a |
| INBio_4504N | Passalidae | PDA agar | Filamentous | Irregular | Convex | 39 - Cinnamon | Myrothecium sp. W MG-2013 | KF723006 | 1212 | 100 | 0 | 2143 | 98.6 | Aerated municipal sewage sludge |
| INBio_4504S | Passalidae | PDA agar | Entire | Circular | Umbonate | 30 - Olive | Exophiala pisciphila | AF050272 | 1261 | 100 | 0 | 1912 | 94.37 | n/a |
| INBio_4504T | Passalidae | PDA agar | Undulate | Irregular | Umbonate | 33 - Cinnamon-brown | Linkosia multiseptum | DQ408572 | 833 | 66 | 0 | 1432 | 97.72 | n/a |
| INBio_4504U | Passalidae | PDA agar | Entire | Circular | Umbonate | 271 - White | Dacrymyces chrysospermus | AF287855 | 851 | 71 | 0 | 1317 | 94.71 | n/a |
| INBio_4504V | Passalidae | PDA agar | Erose | Circular | Umbonate | 119D - Drab-gray | Nectria berolinensis | HM534893 | 1216 | 100 | 0 | 1760 | 93.09 | Host=*Ribes sanguineum* |
| INBio_4504W | Passalidae | PDA agar | Entire | Circular | Umbonate | 30 - Olive | Cladophialophora australiensis | EU035402 | 1252 | 100 | 0 | 1903 | 94.33 | n/a |
| INBio_4505I | Substrate related to Passalidae | PDA agar | Erose | Circular | Concavo | 44 - Smoke gray | Hypomyces australasiaticus | FN859428 | 1235 | 100 | 0 | 2159 | 98.38 | n/a |
| INBio_4505J | Substrate related to Passalidae | PDA agar | Filamentous | Filamentous | Raised | 92 - Pale horn | uncultured Ascomycota | EU490011 | 1217 | 100 | 0 | 1792 | 93.34 | Soil |
| INBio_4505K | Substrate related to Passalidae | PDA agar | Entire | Circular | Convex | 56 - Straw yellow | Mucor sp. BESC196c | KC007161 | 953 | 76 | 0 | 1480 | 94.96 | Plant roots. Host=*Populus trichocarpa* |
| INBio_4505L | Substrate related to Passalidae | PDA agar | Filamentous | Circular | Convex | 271 - White | Anthostomella leucospermi | EU552100 | 1215 | 100 | 0 | 2021 | 96.79 | n/a |
| INBio_4505M | Substrate related to Passalidae | PDA agar | Filamentous | Irregular | Raised | 27 - Drab | Pseudallescheria apiosperma | FJ345358 | 1258 | 99 | 0 | 2200 | 98.33 | n/a |
| INBio_4505N | Substrate related to Passalidae | PDA agar | Entire | Circular | Convex | 271 - White | Elaphocordyceps ophioglossoides | AB027367 | 1216 | 100 | 0 | 2078 | 97.62 | n/a |
| INBio_4505O | Substrate related to Passalidae | PDA agar | Filamentous | Circular | Convex | 271 - White | Talaromyces pinophilus | HM469418 | 1219 | 100 | 0 | 2178 | 98.93 | n/a |
| INBio_4505S | Substrate related to Passalidae | PDA agar | Entire | Circular | Convex | 30 - Olive | Capronia coronata | AF050242 | 1255 | 100 | 0 | 2010 | 95.7 | n/a |
| INBio_4506K | Passalidae | PDA agar | Filamentous | Filamentous | Convex | 92 - Pale horn | Hypocrea lutea | AB027384 | 1217 | 100 | 0 | 2231 | 99.75 | n/a |
| INBio_4506L | Passalidae | PDA agar | Filamentous | Filamentous | Raised | 17 - Spectrum orange | Nectria cinnabarina | HM534894 | 1223 | 100 | 0 | 1958 | 95.75 | Host=*Frangula alnus* |
| INBio_4506M | Passalidae | PDA agar | Erose | Circular | Convex | 271 - White | Metarhizium anisopliae | FJ755244 | 1235 | 100 | 0 | 2241 | 99.43 | n/a |
| INBio_4506N | Passalidae | PDA agar | Filamentous | Circular | Umbonate | 44 - Smoke gray | Leptographium gibbsii | JN940177 | 1186 | 94 | 0 | 1472 | 89.46 | n/a |
| INBio_4506O | Passalidae | PDA agar | Filamentous | Circular | Convex | 84 - Medium neutral gra | Leptographium gibbsii | JN940177 | 1178 | 93 | 0 | 1500 | 90.07 | n/a |
| INBio_4506P | Passalidae | PDA agar | Entire | Circular | Convex | 271 - White | Metarhizium anisopliae | AB027383 | 1254 | 100 | 0 | 1847 | 93.62 | n/a |
| INBio_4506Q | Passalidae | PDA agar | Filamentous | Circular | Convex | 46 - Olive-green (basic | Metarhizium anisopliae | FJ755244 | 1234 | 100 | 0 | 2244 | 99.51 | n/a |
| INBio_4506R | Passalidae | PDA agar | Filamentous | Circular | Convex | 44 - Smoke gray | Leptographium gibbsii | JN940177 | 1186 | 94 | 0 | 1472 | 89.46 | n/a |
| INBio_4506S | Passalidae | PDA agar | Erose | Circular | Convex | 42 - Olive-gray | Ochrocladosporium frigidarii | FJ755255 | 1224 | 100 | 0 | 1899 | 94.93 | n/a |
| INBio_4506T | Passalidae | PDA agar | Erose | Circular | Convex | 271 - White | Neonectria punicea | HM534901 | 1220 | 100 | 0 | 1842 | 94.1 | Host=*Frangula alnus* |
| INBio_4507E | Passalidae | PDA agar | Filamentous | Filamentous | Raised | 92 - Pale horn | uncultured Ascomycota | EU490011 | 1221 | 100 | 0 | 1805 | 93.53 | Soil |
| INBio_4507F | Passalidae | PDA agar | Filamentous | Circular | Convex | 37 - Antique brown | uncultured ectomycorrhizal fungus | AM412280 | 1222 | 100 | 0 | 1687 | 91.82 | Host=*Vateriopsis seychellarum* |
| INBio_4507G | Passalidae | PDA agar | Filamentous | Filamentous | Convex | 123C - Yellow ocher | Nectria cinnabarina | HM534894 | 1224 | 100 | 0 | 1954 | 95.67 | Host=*Frangula alnus* |
| INBio_4507H | Passalidae | PDA agar | Filamentous | Circular | Convex | 86 - Pale neutral gray | Leptographium gibbsii | JN940177 | 1187 | 94 | 0 | 1483 | 89.64 | n/a |
| INBio_4508E | Substrate related to Passalidae | PDA agar | Filamentous | Filamentous | Convex | 92 - Pale horn | Hypocrea lutea | AB027384 | 1217 | 100 | 0 | 2231 | 99.75 | n/a |
| INBio_4508F | Substrate related to Passalidae | PDA agar | Filamentous | Circular | Convex | 45 - Smoke gray | Leptographium gibbsii | JN940177 | 1186 | 94 | 0 | 1472 | 89.46 | n/a |
| INBio_4509G | Passalidae | PDA agar | Filamentous | Filamentous | Raised | 92 - Pale horn | Amorphotheca resinae | EU040230 | 1218 | 100 | 0 | 1797 | 93.51 | n/a |
| INBio_4509I | Passalidae | PDA agar | Entire | Circular | Umbonate | 271 - White | Elaphocordyceps ophioglossoides | AB027367 | 1216 | 100 | 0 | 2078 | 97.62 | n/a |
| INBio_4509J | Passalidae | PDA agar | Entire | Circular | Convex | 44 - Smoke gray | Chaetosphaeria vermicularioides | AF178550 | 1199 | 100 | 0 | 2067 | 97.83 | n/a |
| INBio_4509K | Passalidae | PDA agar | Filamentous | Filamentous | Raised | 30 - Olive | Amorphotheca resinae | EU040230 | 1216 | 100 | 0 | 1742 | 92.76 | n/a |
| INBio_4509L | Passalidae | PDA agar | Filamentous | Filamentous | Raised | 271 - White | Elaphocordyceps ophioglossoides | AB027367 | 1216 | 100 | 0 | 2078 | 97.62 | n/a |
| INBio_4510C | Passalidae | PDA agar | n/a | n/a | n/a | n/a | Scheffersomyces stipitis CBS 6054 | CP000497 | 1255 | 100 | 0 | 2300 | 99.76 | n/a |
| INBio_4510H | Passalidae | PDA agar | Filamentous | Filamentous | Raised | 92 - Pale horn | Amorphotheca resinae | EU040230 | 1218 | 100 | 0 | 1797 | 93.51 | n/a |
| INBio_4510J | Passalidae | PDA agar | Entire | Circular | Convex | 44 - Smoke gray | Chaetosphaeria vermicularioides | AF178550 | 1198 | 100 | 0 | 2071 | 97.91 | n/a |
| INBio_4511E | Substrate related to Passalidae | PDA agar | Filamentous | Filamentous | Raised | 92 - Pale horn | Amorphotheca resinae | EU040230 | 1218 | 100 | 0 | 1797 | 93.51 | n/a |
| INBio_4511I | Substrate related to Passalidae | PDA agar | Entire | Circular | Umbonate | 30 - Olive | Ramichloridium anceps | AF050285 | 1257 | 100 | 0 | 2001 | 95.54 | n/a |
| INBio_4512F | Passalidae | PDA agar | Undulate | Circular | Raised | 30 - Olive | Chaetosphaeria vermicularioides | AF178550 | 1198 | 100 | 0 | 2001 | 96.83 | n/a |
| INBio_4512G | Passalidae | PDA agar | Filamentous | Filamentous | Convex | 271 - White | Leptographium gibbsii | JN940177 | 1186 | 94 | 0 | 1467 | 89.38 | n/a |
| INBio_4512H | Passalidae | PDA agar | Filamentous | Filamentous | Raised | 27 - Drab | Verticillium dahliae | AF104926 | 1206 | 100 | 0 | 1810 | 93.95 | n/a |
| INBio_4512I | Passalidae | PDA agar | Filamentous | Filamentous | Raised | 30 - Olive | Leptographium gibbsii | JN940177 | 1177 | 93 | 0 | 1493 | 89.97 | n/a |
| INBio_4512J | Passalidae | PDA agar | Filamentous | Filamentous | Raised | 30 - Olive | Chaetosphaeria vermicularioides | AF178550 | 1198 | 100 | 0 | 2001 | 96.83 | n/a |
| INBio_4513E | Passalidae | PDA agar | Filamentous | Filamentous | Raised | 92 - Pale horn | uncultured Ascomycota | EU490011 | 1220 | 100 | 0 | 1810 | 93.61 | Soil |
| INBio_4513F | Passalidae | PDA agar | Undulate | Circular | Raised | 30 - Olive | Chaetosphaeria vermicularioides | AF178550 | 1198 | 100 | 0 | 2001 | 96.83 | n/a |
| INBio_4513G | Passalidae | PDA agar | Filamentous | Circular | Umbonate | 30 - Olive | Leptographium gibbsii | JN940177 | 1177 | 93 | 0 | 1493 | 89.97 | n/a |
| INBio_4513H | Passalidae | PDA agar | Undulate | Circular | Raised | 30 - Olive | Chaetosphaeria vermicularioides | AF178550 | 1199 | 100 | 0 | 1995 | 96.75 | n/a |
| INBio_4513I | Passalidae | PDA agar | Erose | Circular | Umbonate | 271 - White | Dacrymyces chrysospermus | AF287855 | 852 | 71 | 0 | 1312 | 94.6 | n/a |
| INBio_4513J | Passalidae | PDA agar | Erose | Circular | Umbonate | 6 - Salmon | Umbilicaria esculenta | EU534208 | 908 | 88 | 0 | 1269 | 92.07 | n/a |
| INBio_4513K | Passalidae | PDA agar | Entire | Circular | Convex | 30 - Olive | Chaetothyriales sp. M-Cre1-5 | HQ634635 | 1256 | 100 | 0 | 2174 | 98.01 | Host=*Crematogaster* sp. |
| INBio_4513L | Passalidae | PDA agar | Erose | Circular | Umbonate | 6 - Salmon | Umbilicaria esculenta | EU534208 | 908 | 88 | 0 | 1258 | 91.85 | n/a |
| INBio_4514C | Substrate related to Passalidae | PDA agar | Filamentous | Circular | Raised | 26 - Clay | Leptographium gibbsii | JN940177 | 1186 | 94 | 0 | 1472 | 89.46 | n/a |
| INBio_4514D | Substrate related to Passalidae | PDA agar | Erose | Circular | Umbonate | 30 - Olive | Ramichloridium anceps | AF050285 | 1258 | 100 | 0 | 1969 | 95.15 | n/a |
| INBio_4514E | Substrate related to Passalidae | PDA agar | Filamentous | Irregular | Umbonate | 30 - Olive | Phaeoisaria clematidis | EU552148 | 1213 | 100 | 0 | 2073 | 97.61 | Host=*Protea neriifolia* |
| INBio_4515F | Passalidae | PDA agar | Filamentous | Filamentous | Raised | 92 - Pale horn | Trichoderma asperellum | KF723005 | 1225 | 100 | 0 | 2239 | 99.67 | Aerated municipal sewage sludge |
| INBio_4515G | Passalidae | PDA agar | Filamentous | Filamentous | Raised | 92 - Pale horn | Trichoderma asperellum | KF723005 | 1225 | 100 | 0 | 2239 | 99.67 | Aerated municipal sewage sludge |
| INBio_4515H | Passalidae | PDA agar | Filamentous | Filamentous | Convex | 271 - White | Elaphocordyceps inegoensis | AB027368 | 1207 | 100 | 0 | 2098 | 98.09 | n/a |
| INBio_4515I | Passalidae | PDA agar | Curled | Filamentous | Convex | 271 - White | Myrothecium sp. W MG-2013 | KF723006 | 1213 | 100 | 0 | 2134 | 98.43 | Aerated municipal sewage sludge |
| INBio_4515K | Passalidae | PDA agar | Filamentous | Filamentous | Raised | 92 - Pale horn | Fusarium solani | FJ345352 | 1224 | 100 | 0 | 2097 | 97.63 | n/a |
| INBio_4515L | Passalidae | PDA agar | Filamentous | Irregular | Convex | 271 - White | Elaphocordyceps ophioglossoides | AB027367 | 1217 | 100 | 0 | 2074 | 97.53 | n/a |
| INBio_4515M | Passalidae | PDA agar | Entire | Circular | Convex | 55 - Spectrum yellow | Bionectria cf. ochroleuca CBS 113336 | EU552110 | 1216 | 100 | 0 | 2141 | 98.52 | Host=*Protea nitida* |
| INBio_4515N | Passalidae | PDA agar | Filamentous | Filamentous | Umbonate | 30 - Olive | Leptographium gibbsii | JN940177 | 1188 | 94 | 0 | 1489 | 89.73 | n/a |
| INBio_4516F | Passalidae | PDA agar | Filamentous | Filamentous | Raised | 92 - Pale horn | uncultured Ascomycota | EU490011 | 1220 | 100 | 0 | 1810 | 93.61 | Soil |
| INBio_4516G | Passalidae | PDA agar | Filamentous | Filamentous | Raised | 92 - Pale horn | uncultured Ascomycota | EU490011 | 1221 | 100 | 0 | 1805 | 93.53 | Soil |
| INBio_4516H | Passalidae | PDA agar | Filamentous | Circular | Raised | 53 - Buff-yellow | Nectria cinnabarina | HM534894 | 1223 | 100 | 0 | 1958 | 95.75 | Host=*Frangula alnus* |
| INBio_4516I | Passalidae | PDA agar | Filamentous | Filamentous | Raised | 39 - Cinnamon | Nectria cinnabarina | HM534894 | 1223 | 100 | 0 | 1958 | 95.75 | Host=*Frangula alnus* |
| INBio_4516J | Passalidae | PDA agar | Curled | Irregular | Umbonate | 85 - Light Neutral gray | Leptographium gibbsii | JN940177 | 1177 | 93 | 0 | 1504 | 90.14 | n/a |
| INBio_4516R | Passalidae | PDA agar | Entire | Circular | Convex | 30 - Olive | Verticillium dahliae | AF104926 | 1233 | 100 | 0 | 1882 | 94.4 | n/a |
| INBio_4517E | Substrate related to Passalidae | PDA agar | Filamentous | Filamentous | Raised | 92 - Pale horn | Trichoderma asperellum | KF723005 | 1225 | 100 | 0 | 2239 | 99.67 | Aerated municipal sewage sludge |

*n/a= not available
